# Photocatalytic overall water splitting endowed by modulation of internal and external energy fields

**DOI:** 10.1039/d4sc05065g

**Published:** 2024-10-04

**Authors:** Wenhao Zhao, Haijun Chen, Jinqiang Zhang, Paul J. Low, Hongqi Sun

**Affiliations:** a School of Molecular Sciences, The University of Western Australia 35 Stirling Highway Perth Western Australia 6009 Australia hongqi.sun@uwa.edu.au; b Jiangsu Key Laboratory of Process Enhancement and New Energy Equipment Technology, School of Mechanical and Power Engineering, Nanjing Tech University Nanjing 211816 Jiangsu China; c School of Chemical Engineering, The University of Adelaide North Terrace Adelaide SA 5005 Australia

## Abstract

The pursuit of sustainable and clean energy sources has driven extensive research into the generation and use of novel energy vectors. The photocatalytic overall water splitting (POWS) reaction has been identified as a promising approach for harnessing solar energy to produce hydrogen to be used as a clean energy carrier. Materials chemistry and associated photocatalyst design are key to the further improvement of the efficiency of the POWS reaction through the optimization of charge carrier separation, migration and interfacial reaction kinetics. This review examines the latest progress in POWS, ranging from key catalyst materials to modification strategies and reaction design. Critical analysis focuses on carrier separation and promotion from the perspective of internal and external energy fields, aiming to trace the driving force behind the POWS process and explore the potential for industrial development of this technology. This review concludes by presenting perspectives on the emerging opportunities for this technology, and the challenges to be overcome by future studies.

## Abstract 2

The challenges of photocatalytic overall water splitting (POWS), including thermodynamic constraints, carrier generation and transport, and macroscopic reaction kinetics, are addressed by both intrinsic and externally applied energy fields.

## Graphical abstract

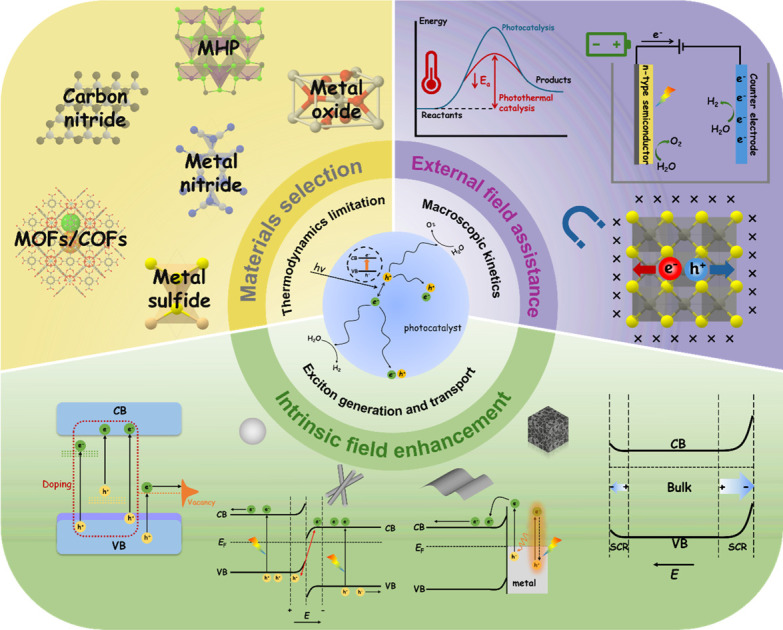

## 1. Introduction

The energy needs of society required to maintain and expand the living standards and well-being of a growing human population is a major, urgent and global problem facing mankind in the 21st century. Moreover, this challenge must be met without excessive use of fossil fuels, as carbon emissions are believed to be the main driver of deleterious climate change. Whilst semiconductor solar cells and wind turbines provide a means to directly generate electricity, it is necessary to convert the resulting energy to suitable carriers for storage and transport to points of need. Hydrogen has been identified as one of the ideal energy vectors, offering advantages such as high calorific value, good combustion performance, excellent thermal conductivity, non-toxicity, zero emission, and capacity to be stored in diverse forms for transport and release.^1^

Hydrogen can obtained by splitting water, the most abundant natural resource on Earth, by electrolysis or through the photocatalytic water splitting, a process first demonstrated at a TiO_2_ electrode in the 1970s.^2^ The use of electricity generated from renewable sources or direct photocatalytic reactions to generate ‘green’ hydrogen provides a route to renewable energy storage, whilst simultaneously curbing greenhouse gas emissions and fostering sustainability of energy supply. In turn, the use of hydrogen as an energy source propels technological advancements in furnace and fuel cell technologies, fortifies global energy security, and enhances global economic opportunities.^3–6^ Moreover, the integration of hydrogen into existing infrastructure, such as natural gas pipelines, facilitates a gradual transition to cleaner energy sources without the need for substantial system overhauls.

However, despite the immense promise, electrolysis of water to produce hydrogen at scale necessitates the use of inert metal electrodes, such as platinum, to avoid deleterious effects of corrosion in the highly oxidising environment of the electrolytic cell. Photocatalytic overall water splitting (POWS) using semiconductor-based materials to produce hydrogen remains at the fundamental research stage with many limitations to industrial production of hydrogen including a low light harvesting efficiency and hydrogen yield remaining to be overcome before and applications of this technology can be realised.^7^ However, the scarcity of platinum and the potential to convert solar energy and water directly to green hydrogen *via* the POWS has driven the search for efficient photocatalysts and design strategies to improve the efficiency of the POWS efficiency in order to make solar-driven hydrogen production practical for industry applications.

The key to improving the efficiency of photocatalytic water splitting is to suppress the recombination of the photogenerated electron/hole pairs in the photocatalyst before the charge-separated state can participate in an effective photocatalytic reaction.^8^ To address this problem, many strategies have been explored to modify the catalyst materials, for example, surface modification of the semiconductor photocatalyst with metal co-catalyst,^9^ the efficiency of which can be further accentuated through the use of nanostructured mixed-metal co-catalysts.^10^ Recognising that the hydrogen evolution reaction runs concurrently with the water oxidation reaction in the POWS process, noble metal oxides, such as RuO_2_ (ref. 11) and IrO_2_,^12^ can be used as excellent water oxidation co-catalysts to improve total photocatalytic activity. Furthermore, systems such as the Rh@Cr_2_O_3_ surface core–shell structure co-catalyst can be designed to inhibit the reverse reaction between H_2_ and O_2_.^13^

The use of two or more materials within the catalyst naturally leads to heterojunctions at the material interfaces. At the interface of the resulting heterostructures, the relative band gaps of the materials can be described as: (a) a straddling gap (type I) where the smaller band gap of one material falls within the larger band gap of the other; (b) a staggered gap (type II) where the band gaps are offset, with the conduction or valence band of one material falling into the band gap of the other; (c) a Z-scheme, which is variation of the type II staggered heterojunction, in which upon contact, electrons flow, with or without the aid of a redox mediator, from the material with the higher Fermi energy to the accumulate in the conduction band of the other whilst holes flow in the reverse direction until the Fermi energies are equilibrated and generating an internal electric field as a consequence of charge separation; and (d) a step or S-scheme, which is a further development of the Z-scheme, in which a material with a high lying conduction band (*i.e.* high reduction ability) is placed in contact with a second material with a low-lying valence band (*i.e.* high oxidation ability) resulting in regions of high electron accumulation and electron depletion near the interface, bending of the band structure and a pronounced internal electric field. Each of these heterojunctions can promote the separation of photogenerated carriers,^14–17^ and all involve the establishment of built-in electric fields. Moniz *et al.* have provided a comprehensive review of visible light-driven heterojunction catalysts, encompassing nearly the entire family of heterojunctions.^15^ In exemplary work, Zhou *et al.* have achieved a solar-to-hydrogen (STH) efficiency of approximately 9.2% with redox-modified InGaN/GaN nanowires and 6.2% in a large-scale POWS system under a high natural sunlight intensity of around 16 070 mW cm^−2^, thereby demonstrating the practical applicability of solar hydrogen production technology beyond laboratory settings.^6^ With the expansion of material systems, materials with additional properties, including surface plasmon resonances,^18^ pyroelectric behaviour,^19^ and ferroelectric character,^20^ have been developed and applied to POWS technology. The working principles of these catalysts can be traced to the establishment of internal electric fields. Understanding the principles of carrier separation and mobility that arise because of this internal distribution of charge are critical to design strategies that can extend the lifetime of photogenerated carriers.

The internal electric and thermal fields within materials and applied external energy fields (*e.g.*, thermal, electric, and magnetic fields) can significantly affect the behaviour of photo-excited charge carriers in catalysts and catalytic throughput.^21–24^ As implied by the description of heterojunctions above, internal electric fields typically arise from the polarization of uneven charge distributions across various component layers,^25^ and numerous studies have focused on heterojunction-type internal electric fields to influence the dynamic behaviour of photogenerated excitons and improve the performance of POWS. For instance, Takata *et al.* enhanced the distribution of electric fields within a crystal by selectively depositing separate co-catalysts for the hydrogen and oxygen evolution reactions on different crystal facets of a modified Al-doped strontium titanate semiconductor photocatalyst, resulting in an enhanced rate of forward (productive) charge transfer events and achieving an internal quantum efficiency approaching unity.^26^ Similarly, albeit through a physical mechanism rather than an intrinsic change in materials structure through introduction of different heterojunctions, Li *et al.* demonstrated effective seawater splitting using N-doped TiO_2_ with selective accumulation of electrolyte ions of opposite charge from the reaction medium on different photopolarized crystal facets, thereby and prolonging the charge-carrier lifetime by a factor of five.^27^

Moreover, strategies aiming at modulating the band structure of semiconductors, such as doping or introduction of defects, have recently been shown to create non-centrosymmetric structures within semiconductor catalysts, thereby inducing spontaneous polarization and generating internal electric fields.^28^ A notable example is the work reported by Li *et al.*, who doped carbon into Bi_3_O_4_Cl nanosheets, resulting in a 126-fold enhancement of its internal electric field and achieving a bulk charge separation efficiency of 80%.^29^ Analogous to the effect of doping that leads to uneven local electron distributions, defects can also disrupt or alter the perfect periodic arrangement of atoms or molecules in crystalline materials, thereby modifying the local electronic states or space charge regions in response to changes in the internal electric field.^30^

Furthermore, traditional morphology control strategies aiming at enhancing the light-harvesting capabilities of photocatalysts have recently been shown to significantly facilitate internal field enhancement, with the tip effect being the most frequently documented phenomenon.^31^ The tip effect pertains to the concentration of energy forms, including electrons, photons, and magnetic fields, at regions of high curvature, which results in the amplification of local electric, thermal, and magnetic fields.^32^ This phenomenon is also referred to the “wrinkle” effect in semiconductor nanomaterials, and which will be elaborated upon in subsequent sections of this review. Notably, the wrinkle effect is most prominently observed in metallic nanomaterials, commonly recognized as the localized surface plasmon resonance (LSPR) enhancement effect.^33^ Intriguingly, the LSPR effect in metals not only contributes to the enhancement of the local internal electric field but is also associated with a thermal effect that can elevate local temperatures, thereby enhancing the apparent activity of the catalyst to a certain degree.^34^ For instance, Ha *et al.* illustrated that hot electrons produced by Au nanorods can be effectively transferred to TiO_2_ situated at the tips of the nanorods for the purposes of storage and utilization. This process is facilitated by the LSPR effect of the Au nanorods, resulting in selective photoreduction and photooxidation of water occurring at the tip TiO_2_ and the sides of the Au nanorods, respectively.^35^

In the context of external field-assisted POWS, the mechanism of field enhancement is particularly well-defined, especially concerning external electric and magnetic fields. POWS technology is rooted in photoelectrochemical reactions, where the mechanism of the external electric field aligns with that of traditional electrochemical reactions, while the influence of the magnetic field primarily draws upon the Lorentz effect.^19^ Both mechanisms play a significant role in the overall directional separation of macroscopic carriers. In contrast, the impact of the external thermal field on carrier dynamics remains a subject of debate. One perspective posits that lattice vibrations induced by temperature increases can lead to electron-phonon scattering.^36^ However, in the case of thermodynamically uphill reactions such as water splitting, a macroscopic increase in temperature is likely to result in a reduction of the reaction barrier. Consequently, modulation of these energy fields becomes crucial for optimizing the energy consumption, improving the light utilization efficiency, and promoting the hydrogen production. This approach is thus promising in harnessing full-spectrum sunlight for scalable green hydrogen generation.

There are a number of excellent review articles that address various of these aspects of the POWS. For instance, Sun *et al.* have detailed the advantages of metal–organic frameworks (MOFs) as modularly assembled solids that allow elucidation of several of the structure–performance relationships that are important in POWS and CO_2_ reduction.^37^ Zhao *et al.* have reviewed transition metal-based co-catalysts for water splitting in recent years,^38^ while Tao *et al.* have highlighted materials design strategies to increase the charge separation within particulate photocatalysts, including the introduction of surface junctions, spatial charge separation between facets, and polarity-induced charge separation.^39^ Li *et al.* have described and summarised the effects of external thermal gradients, magnetic fields, microwave radiation and ultrasound waves on a variety of photocatalysts and the impact on the reactions that they promote.^23^ However, the effects of band structure, morphology control, heterojunctions, surface/interface modification and the range of external fields and stimuli on the internal charge generation, transport and surface chemistry of the photocatalysts that promote the POWS have not been thoroughly reviewed, despite the insight that such a collated description would provide for future catalyst designs.

In this review, we first discuss the challenges of POWS from three perspectives: thermodynamics; charge carrier generation and transport; and macroscopic reaction kinetics. On this basis, the corresponding solutions are proposed from three fundamental aspects: material composition and structure; internal fields; and the application of external stimuli. Specifically, the review focuses on the different types of semiconductor materials that have been applied as photocatalysts for the POWS, with the effects of the semiconductor bandgap, and the mainstream catalyst modification strategies on the photoexciton generation and transport, and the chemical aspects of the overall water splitting reaction mediated at the surface. The impact of these higher-level concepts is interpreted from the perspective of the internal intrinsic electric/polarization field within these materials and the influence of an applied external field. The effects of different reaction types on the reaction kinetics of different materials are also summarized and discussed. Finally, the limitations of contemporary POWS systems are summarized, and directions for future development are proposed.

## 2. Fundamentals and challenges of POWS

**Fig. 1:**
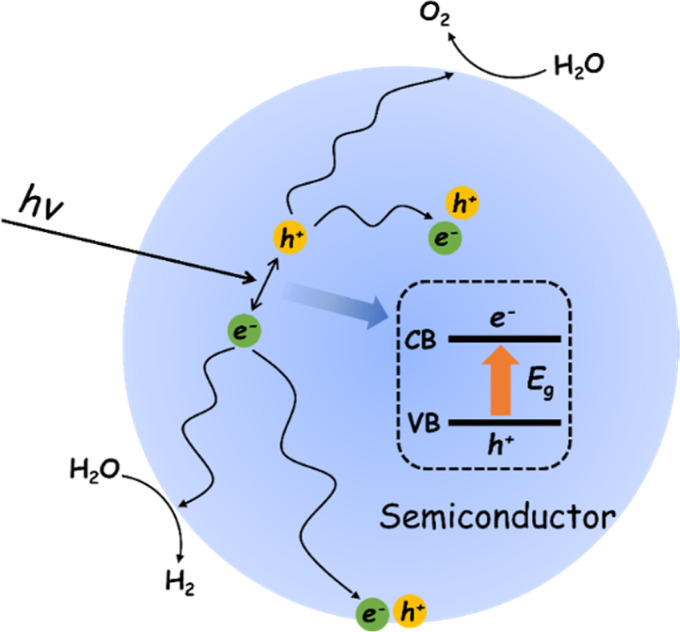
Physical/chemical processes of the photocatalytic overall water splitting reaction.

The POWS reaction on a semiconductor photocatalyst can be broadly considered in terms of three general processes (Fig. 1).^40^ Impact of light of energy greater than the semiconductor bandgap (*E*_g_) results in promotion of an electron from the top of the valence band (VB) to the conduction band (CB). The resulting hole in the VB (h^+^) and electron in the CB (e^−^) can recombine in the bulk, with the net result that the absorbed light energy has simply been converted into heat energy, or emitted as a photon of lower energy through fluorescence. If the photogenerated charges diffuse away from each other before recombination, the resulting charge separated pair may migrate to the surface where they may again recombine or promote the coupled reduction and oxidation steps necessary to achieve the overall water splitting reaction to give H_2_ and O_2_.^41^

However, beneath these elementary descriptive steps, the POWS combines very complex physical and chemical process, which brings about great challenges to achieve high efficiencies. The common problem of a high carrier recombination rate within the bulk is compounded by the surface state and environment that strongly influence charge recombination at the photocatalyst surface.^42^ Further complicating the model, the processes of charge separation, migration and the surface reactions with substrates cannot be independently optimised as they are inherently related to each other.^43^ In other words, the imbalance between the rate of charge migration to the rate of the chemical steps involved in the reaction at which take place at sites on the surface with specific structure governed by the nature of the reactants and products will also lead to a low overall reaction efficiency.

In addition, in thermodynamic terms, the splitting of water into H_2_ and O_2_ in an electrolytic cell is an energy-consuming reaction, theoretically requiring 1.23 eV (Δ*G* = + 237 kJ mol^−1^) of energy (eqn (1)) based on the redox potential of the water splitting reaction (H^+^/H_2_, 0 V and O_2_/H_2_O, 1.23 V *vs.* normal hydrogen electrode at pH = 0).^44^ In contrast, the direct reverse reaction, *i.e.*, the combination of H_2_ and O_2_ to reform water, is highly exothermic and more thermodynamically favourable than the POWS reaction.^45^ Therefore, many studies have introduced sacrificial redox agents into the reaction system to quench either the hydrogen evolution reaction (HER) or oxygen evolution reaction (OER) and avoid the recombination of H_2_ and O_2_ products. However, these sacrificial half-reactions, which are thermodynamically downhill processes, are accompanied by a reduction in Gibbs free energy, resulting in a waste of photon energy.^46^ Developing photocatalysts that do not require these reagents to achieve productive overall water splitting remains a great challenge.

1 2H_2_O → 2H_2_ + O_2_, Δ*G* = +237 kJ mol^−1^

The POWS process is also governed by carrier dynamics. The generation, separation, and migration of photogenerated charges to the catalyst surface occur on the timescale of femtoseconds to nanoseconds, while the subsequent redox reactions on the surface take much longer (microseconds to seconds)^47^ and are generally considered the rate-limiting step of the entire photocatalytic reaction. Since the effective amount of photogenerated h^+^ is larger than that of e^−^,^48^ the migration rates of the two in the catalyst bulk are also different, leading to the incompatibility of the OER and HER reaction rates. In addition, the larger overpotential of the OER relative to the HER and the greater affinity of O_2_ for most catalyst surfaces^49^ results in a greater thermodynamic driving force to promote the OER.

Therefore, it can conclude that the current challenges facing POWS mainly lie in the following three areas:

(a) The thermodynamic limitation of POWS reaction, that is, the endothermic nature of the forward reaction and reverse exothermic characteristics, hinders the coexistence of H_2_ and O_2_, and poses a challenge to the redox potential of semiconductor catalysts, indicating that the development of photocatalysts with high excited state redox potentials necessary to overcome the overpotentials and provide the net driving force for the reaction is still a research theme in this field.

(b) The incompatibility between the separation and transport of photogenerated carriers within the bulk and the facile charge recombination reactions on semiconductors surfaces are among the main reasons for the low efficiency of the POWS reaction. Internal field enhancement combined with surface modification can be an effective means to improve charge separation whilst increasing the dispersion and randomness of surface reactions, thereby reducing carrier pair self-annihilation.

(c) The macroscopic kinetics of the charge carriers is a core limitation to the surface-catalysed reactions, due to the longer time associated with photoexcitons acting on reactants compared to the microscopic separation and migration processes. The apparent reaction rate and mass transfer efficiency can be improved by upgrading the reaction system to improve the surface catalytic response, with assistance from the application of an external field.

## 3. Basic material principles for POWS

### 3.1 Host semiconductor nanomaterials

**Fig. 2:**
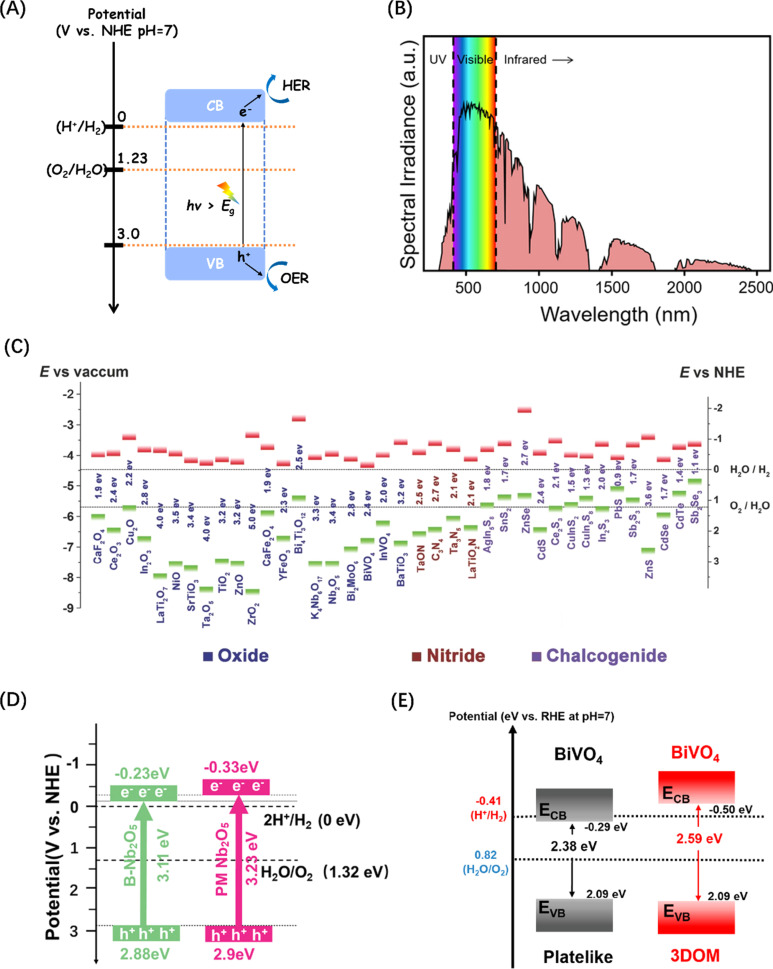
(A) Schematic energy level diagram of the POWS reaction of semiconductor photocatalysts. (B) Standard solar radiation spectrum at the Earth surface at sea level. Reproduced from ref. 50 with permission from American Chemical Society, copyright 2022. (C) Band structure comparison of metal oxides commonly used in POWS. Reproduced from ref. 51 with permission from John Wiley and Sons, copyright 2015. (D) Energy band structure diagrams for two types of Nb_2_O_5_. Reproduced from ref. 52 with permission from John Wiley and Sons, copyright 2024. (E) Energy band structure diagrams for two types of BiVO_4_. Reproduced from ref. 53 with permission from American Chemical Society, copyright 2021.

In order to realize the simultaneous occurrence of the electron-driven water reduction reaction and the hole-driven water oxidation reaction, the band edge positions of a semiconductor are critical. Effective reaction requires the reduction electrode potential and oxidation electrode potential of water fall within the VB and CB potentials of the semiconductor (Fig. 2A), ensuring that the potential of the conduction band minimum (CBM) where the photogenerated e^−^ are located is more negative than the H^+^/H_2_ energy level (0 V, eqn (2)), and the potential of the valence band maximum (VBM) where the photogenerated h^+^ are located is more positive than the O_2_/H_2_O energy level (1.23 V, eqn (3)).^39^ In addition to the band edge positions, *E*_g_ is another important parameter that shapes the activity of a semiconductor, because the bandgap width of the semiconductor determines its light absorption threshold and directly affects its utilization of light energy. The wavelength range of solar spectrum that can be observed on the Earth is 295–2500 nm,^54^ of which infrared light accounts for about 53%, visible light for about 44%, and ultraviolet light only for about 3% (Fig. 2B).^50^ Therefore, the semiconductor band gap should be theoretically less than 3.0 eV to improve the utilization of the visible light (>400 nm), according to the relationship (eqn (4)) between the semiconductor band gap and light absorption.^55^

2 2H^+^ + 2e^−^ → H_2_, *E* = 0 V

3 2H_2_O + 4h^+^ → 4H^+^ + O_2_, *E* = 1.23 V

4 *αhv* = (*hv* − *E*_g_)^*n*^ where *n* = 1 or 1/2

Semiconductor-based photocatalytic water splitting technology was developed in early 1970s, marked by the discovery of the process on a TiO_2_ semiconductor single crystal electrode.^2^ Since then, a variety of photocatalysts have been studied and reported. The general principle demonstrated by these photocatalysts is that metal cations with one of two specific electronic structures are active in POWS: transition metal cations with a d^0^ electron configuration (such as Ti^4+^, Zr^4+^, Nb^5+^, Ta^5+^, and W^6+^), and typical metal cations with d^10^ electron configuration (such as Ga^3+^, In^3+^, Ge^4+^, Sn^4+^, and Sb^5+^), while the CBM of the corresponding oxide is mainly contributed by the empty d or sp orbital of the metal cation.^45,46^ Since the VBM consisting of O_2p_ orbitals lies at about 3 V (relative to normal hydrogen electrode (NHE) at pH = 0),^46^ then as long as the potential of the CBM is less than zero (more negative than the H^+^/H_2_ energy level), the bandgap energy of these oxides will be higher than 3 eV. As a result, both d^0^-type and d^10^-type oxides tend to exhibit POWS activity (Fig. 2C).^51^ Recent studies have indicated that the band energies of traditional metal oxide semiconductor materials like Nb_2_O_5_ (ref. 52) and BiVO_4_ (ref. 53) can be tuned by changes to the macroscopic structure, leading to an increased potential for POWS, as shown in Fig. 2(D) and (E). However, the high potential of VB formed by the O_2p_ orbital makes the *E*_g_ theoretically at least 3 eV. However, such band gaps limit the light required to drive POWS activity to the near-ultraviolet range, with no response to visible and near-infrared light, which together account for about 97% of the solar spectrum energy.

**Fig. 3:**
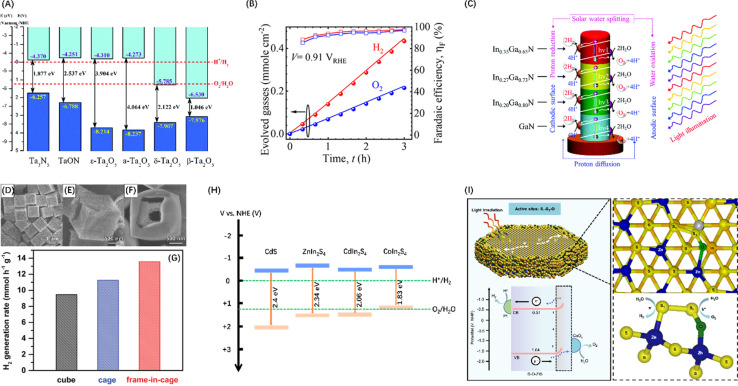
(A) Band diagrams for Ta_2_O_5_, TaON, and Ta_3_N_5_. Reproduced from ref. 57 with permission from Royal Society of Chemistry, copyright 2018. (B) Stoichiometric 2 : 1 evolution of hydrogen and oxygen gases on polycrystalline Ta_3_N_5_ nanorods. Reproduced from ref. 58 with permission from American Chemical Society, copyright 2023. (C) Schematic diagram of POWS process occurring on In_*x*_Ga_1−*x*_N four-band nanowires. Reproduced from ref. 59 with permission from Royal Society of Chemistry, copyright 2019. FESEM images of Cd cubes (D), cage (E), and frame-in-cage (F). (G) Comparison of photocatalytic H_2_ generation rates of CdS nanoparticles with three morphologies. (D–G) Reproduced from ref. 60 with permission from John Wiley and Sons, copyright 2020. (H) Band diagrams for CdS (adapted from ref. 61 with permission from Royal Society of Chemistry, copyright 2019), ZnIn_2_S_4_ (adapted from ref. 62 with permission from John Wiley and Sons, copyright 2021), CdIn_2_S_4_ (adapted from ref. 63 with permission from John Wiley and Sons, copyright 2022), and CoIn_2_S_4_ (adapted from ref. 64 with permission from Elsevier, copyright 2024). (I) Operational mechanism of POWS on DO-ZIS. Reproduced from ref. 65 with permission from Springer Nature, copyright 2024.

To improve the visible light response of photocatalyst candidates, semiconductors with VBM between +1.23 and +3 V have attracted extensive attention. Since N is less electronegative than O, the N_2p_ orbital of metal nitrides form VBs at a less positive potentials than the VBs generated by the O_2p_ orbital in oxides, while the potential of CB is almost unaffected by the introduction of nitrogen.^56^ The crystal and energy band structure of a series of Ta–O–N functional materials have been studied through theoretical calculations, showing that the band gap energy of ε-Ta_2_O_5_, TaON and Ta_3_N_5_ gradually decreases (Fig. 3A), confirming that nitrogen can raise and widen the valence band of Ta–O–N materials.^57^ Polycrystalline Ta_3_N_5_ nanorods with an optimized morphology and crystallization properties attaining near-ideal water splitting with a Faraday efficiency of nearly 100% have also been described (Fig. 3B).^58^ Additionally, metal nitride materials such as Ge_3_N_4_,^66^ GaN,^67^ and InGaN (Fig. 3C)^59^ have also been proven to be efficient in the POWS process.

Apart from nitrides, metal chalcogenides, and particularly metal sulfides, are also favourable candidates as visible light-driven photocatalysts because their VBs typically comprise the p orbitals of S, which have shallower energy levels than that of O_2p_ orbitals.^68^ The most studied metal sulphide semiconductors are ZnS and CdS, between which ZnS has a wide band gap, *i.e.*, 3.6 eV at room temperature.^69^ In contrast, the bandgap energy of CdS is only 2.4 eV,^61^ endowing effective utilization of visible light. An interesting study synthesized three nanostructured forms of CdS materials (Fig. 3D–F) featuring and combining the thin-shell, hollow and frame-like structures that enhance light absorption, minimize distances for the transfer of photogenerated charges to the active surface, and enhance mass transport and explored their POWS potential. These studies confirmed that CdS frame cage particles have supreme activity compared to cubes and cages, with the highest H_2_ generation rate (Fig. 3G).^60^ Additionally, ternary metal sulphides have also attracted much attention because of their higher chemical and photostability than binary metal sulphides. For example, ZnIn_2_S_4_,^62^ CdIn_2_S_4_,^63^ and CoIn_2_S_4_,^64^ have been developed as photocatalysts with efficient visible light response capabilities (Fig. 3H). However, it is still difficult for metal sulphides to completely split H_2_O into H_2_ and O_2_ without carrier sacrificial agents, because S^2−^ in the material can be easily oxidized by h^+^ near the VBM, resulting in self-photocorrosion without the release of O_2_.^70^ Nevertheless, Chong *et al.*^71^ synthesized a superhydrophilic ZnIn_2_S_4_ that can drive photocatalytic pure water splitting and maintain near uniform stability and performance throughout the daytime reaction. It was also confirmed that the charge redistribution caused by defects enhances the activation of water and reduces the surface dynamic barrier. Xin *et al.*^65^ reported a cationic oxygen-doped ZnIn_2_S_4_ (DO-ZIS) induced by lattice distortion and found that the electron-rich S_1_ site in the local structure S_1_–S_2_–O site is favourable for hydrogen adsorption, while the strong charge redistribution property activates a stable oxygen reaction at the electron-deficient S_2_ site, which can avoid the sulphur instability problem common in metal sulphide photocatalysis (Fig. 3I).

**Fig. 4:**
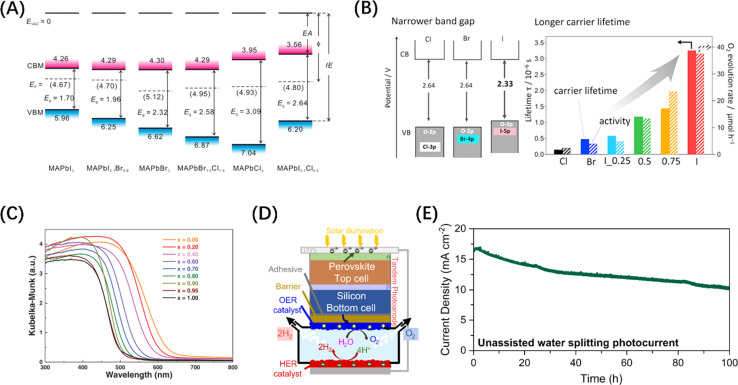
(A) Changes in the energy band levels of MAPbX_3_ after substitution of the *X* position. Reproduced from ref. 73 with permission from American Chemical Society, copyright 2016. (B) Comparison of the band structures of three types of Ba_2_Bi_3_Nb_2_O_11_X and the O_2_ evolution rate over them. Reproduced from ref. 74 with permission from American Chemical Society, copyright 2021. (C) UV-vis diffuse reflectance spectroscopy of La_1−*x*_Ca_*x*_TaO_1+*y*_N_2−*y*_ (0 ≤ *x*, *y* ≤ 1). Reproduced from ref. 75 with permission from John Wiley and Sons, copyright 2020. (D) Schematic diagram of the structure of Si/perovskite composite photochemical cell. (E) 2-Electrode unassisted water-splitting over time in Si/perovskite hybrid photochemical cell. (D and E) Reproduced from ref. 76 with permission from Springer Nature, copyright 2023.

Metal halide perovskite (MHP) materials are ionic crystals with a general chemical formula ABX_3_, in which A site is usually a monovalent cation or ionic group, and B site is a transition metal ion, which ensures that it has a negative CBM.^72^ Generally, the VB band edge of MHP materials can usually be adjusted by the halide anion at the *X* position (Fig. 4A).^73^ For example, it has been reported that Ba_2_Bi_3_Nb_2_O_11_I is not only responsive to a wider range of visible light than chlorides and bromides, but also acts as a stable photocatalyst and can effectively oxidize water (Fig. 4B).^74^ In addition, Wang *et al.* synthesized a perovskite solid solution photocatalyst, La_1−*x*_Ca_*x*_TaO_1+*y*_N_2−*y*_ (0 ≤ *x*, *y* ≤ 1), which also achieved the purpose of regulating the band edge of MHP materials by regulating the ratio of two ions at the A-position (Fig. 4C).^75^ It is worth mentioning that MHP-based solar cells have demonstrated an efficiency higher than 25%,^77^ which makes them an extremely popular material for both solar energy harvesting and photocatalyst research in recent years. Inspired by the structure of perovskite solar cells, an MHP-based photoelectrode system was constructed for water splitting in alkaline electrolytes.^78^ On this basis, improved photoelectrode structures, such as p-type-intrinsic-n-type (p-i-n)^79^ and n-i-p^80^ structures, have also appeared and demonstrated excellent performances. It should be noted that a key reason for introducing MHP materials to electrode structures is that designing perovskite-metal heterostructures is not simple, because noble metal nanoparticles loaded on the surface of MHP nanocrystals tend to agglomerate under steady-state illumination, leading to the loss of noble metal nanostructures and thus affecting the catalytic activity. Despite the widespread adoption of electrode systems, the soft nature of MHPs makes them susceptible to surface transformation or degradation in electrolyte systems.^81^ Therefore, it is particularly important to introduce photoelectrode protection measures while avoiding affecting the mass transfer efficiency. It is exciting to note that Fehr *et al.* designed a halide perovskite-based photochemical cell composed of silicon perovskite monolayers stacked in series, with a peak STH efficiency of 20.8%, and continuous operation for 102 h under AM 1.5 G illumination, showing a new possibility for the development of productive, durable, and competitive solar water splitting technology, as shown in Fig. 4(D) and (E).^76^

**Fig. 5:**
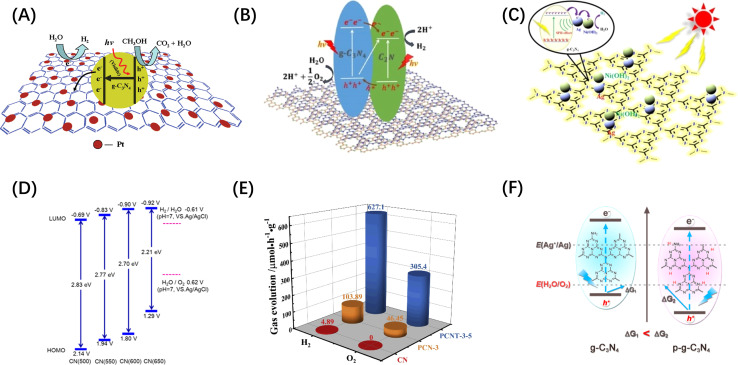
(A) Electron separation and migration enhancement mechanism in graphene/g-C_3_N_4_ composites. Reproduced from ref. 85 with permission from American Chemical Society, copyright 2011. (B) Possible pathways for electron–hole separation in g-C_3_N_4_/C_2_N nanocomposite. Reproduced from ref. 86 with permission from John Wiley and Sons, copyright 2016. (C) Proposed photocatalytic hydrogen production mechanism over Ag/Ni(OH)_2_/g-C_3_N_4_ photocatalyst. Reproduced from ref. 87 with permission from John Wiley and Sons, copyright 2022. (D) Electronic band structure of C_3_N_4_ after heat treatment at different temperatures. Reproduced from ref. 88 with permission from American Chemical Society, copyright 2018. (E) Comparison of POWS products release rates of CN, PCN-3 (P-doped g-C_3_N_4_) and PCNT-3-5. Reproduced from ref. 89 with permission from Elsevier, copyright 2019. (F) Energy band comparison of g-C_3_N_4_ before and after protonation treatment. Reproduced from ref. 90 with permission from American Chemical Society, copyright 2015.

Conjugated polymer nanomaterials (*i.e.* organic semiconductors) have emerged as promising candidates for water splitting in recent years because of their delocalized π-systems and diverse synthetic modularity, which allow them to absorb visible light and to systematically control the electronic and structural properties.^82,83^ In particular, conjugated polymer nanophotocatalysts received more attention after graphitic carbon nitride (g-C_3_N_4_) was demonstrated to be an excellent photocatalyst for visible-light-driven H_2_ production.^84^ For example, graphene,^85^ C_2_N^86^ and Ni(OH)_2_ (ref. 87) modified g-C_3_N_4_ have been demonstrated as visible light responsive photocatalysts, as shown in Fig. 5A–C. DFT studies showed that the CB and VB positions of g-C_3_N_4_ are −1.12 and +1.57 V, respectively.^84^ Similarly, the band gap of g-C_3_N_4_ can be tuned by doping more N in the CN skeleton. Mesoporous carbon nitrides with C_3_N_5_ (ref. 91) and C_3_N_6_ (ref. 92) stoichiometry have been reported to have narrower band gap width than C_3_N_4_. The C/N ratio in carbon nitride can also be controlled by changing the calcination temperature, and hence tune the band gap (Fig. 5D).^88^ However, the OER yield of g-C_3_N_4_-based metal-free photocatalysts is very low and further work is required to design POWS photocatalysts based on these materials.^82^ For example, Ai *et al.*^89^ synthesized a P-doped g-C_3_N_4_/Ti_3_C_2_ composite material (PCNT-3-5), on which the O_2_ evolution rate can be as high as 1527.3 μmol g^−1^, while pristine g-C_3_N_4_ (CN) was not active, as shown in Fig. 5(E). The boosted activity of the complex was ascribed to the construction of heterojunction rather than the improvement of OER activity of g-C_3_N_4_ itself. Chen *et al.* demonstrated that the overall downshifting of the energy band caused by protonation can increase the thermodynamic driving force of g-C_3_N_4_, but a sacrificial redox agent is required in the reaction medium (Fig. 5F).^90^ It concludes that the downward shift requirement of VB and the dependence on sacrificial agents limit the photocatalytic ability of this type of photocatalyst towards POWS.

**Fig. 6:**
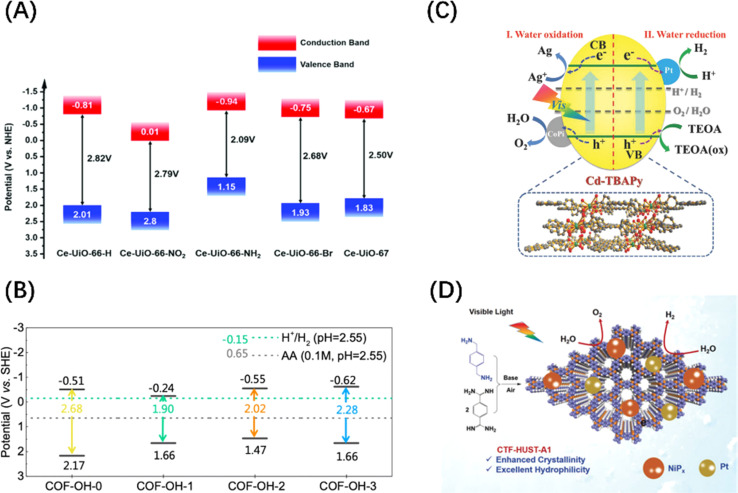
(A) Band structure diagram of UiO66(Zr)-X. Reproduced from ref. 97 with permission from John Wiley and Sons, copyright 2019. (B) Band positions and band gaps of COFs with different numbers of –OH. Reproduced from ref. 98 with permission from Royal Society of Chemistry, copyright 2022. (C) Mechanism diagram of visible light-driven POWS products release on Pt and CoPi co-modified Cd-based MOF. Reproduced from ref. 99 with permission from John Wiley and Sons, copyright 2018. (D) Mechanism diagram of visible-driven POWS on the covalent triazine frameworks (CTFs) co-deposited by Pt and NiP_*x*_. Reproduced from ref. 100 with permission from John Wiley and Sons, copyright 2020.

In addition to the above-mentioned two-dimensional (2D) planar materials, those with a three-dimensional (3D) network topology, such as MOFs and covalent–organic frameworks (COFs) materials, have also been developed as photocatalysts with POWS activity. MOFs utilize coordination chemistry as the driving force to assemble organic or organometallic linkers around metal secondary structural units to form coordination polymers with clear topological structures.^50,93,94^ COFs are periodically arranged porous organic polymers, which are crosslinked into regular structures by linking organic building blocks connected through covalent bonds.^95,96^ Although MOFs and COFs were initially and widely used in gas adsorption and separation because of their macroporous structures and large surface areas, they can both exhibit visible light photocatalytic activity after energy band adjustment. For MOFs materials, the adsorption properties can be tuned by changing the organic ligands, and UiO66(Zr)-X (X = –H, –NH_2_, –NO_2_, and –Br) is a good example (Fig. 6A).^97^ The band gap control of COFs can be achieved by rationally adjusting the building blocks and introducing electron donor–acceptor (D–A) units and π-conjugated systems, such as skeleton group reactions and bulk phase integration and stripping strategies (Fig. 6B).^96,98^ It should be noted that MOF- and COF-based materials have a low carrier mobility and high recombination rate due to the organic linking groups and porous structures, limiting their widespread acceptance. Therefore, the introduction of co-catalyst particles is an indispensable step for enabling these two materials to be effective in POWS, aiming to transfer the traditional catalytic reaction sites from the surface to the interior of the catalyst. This not only spatially separates the carriers but also avoids the recombination of carriers during long-distance transport, as shown in Fig. 6(C) and (D).^99,100^ In contrast to inorganic materials, organic materials exhibit a low dielectric constant and weak non-covalent electronic interactions, which result in the formation of electrostatically bound excitons upon photoexcitation.^101^ For these excitons being effectively utilized as a current or to initiate redox reactions, they must dissociate into free charge carriers within their lifetime. The strength of the electrostatic binding energy of the electron–hole pair is influenced by the magnitude of the Coulomb interaction between the excitons.^102^ From an energy perspective, the strategy of additive modification in organic semiconductor systems aims to enhance the dissociation of excitons by manipulating the energy difference between the charge transfer states of the donor and acceptor components.^103^ Consequently, a significant energy difference between the donor and acceptor is highly desirable, akin to the operational mechanism of the built-in electric field present in Schottky heterojunctions in inorganic semiconductors.

### 3.2 Co-catalysts for hydrogen reduction

While the various semiconductor materials mentioned above have the potential of serving as POWS catalysts, the high carrier recombination rate in the bulk and inefficient surface reactions greatly limit their applications. Especially for the OER reactions, most of the above-mentioned single host catalysts do not have the ability to completely split water to stoichiometrically generate H_2_ and O_2_, because there is a certain difficulty in balancing the band gap width and forming a sufficiently positive VBM. However, it has been proven that modifying the catalyst surface with co-catalysts tailored towards HER and OER activity can significantly improve the POWS performance.^38,104^

**Fig. 7:**
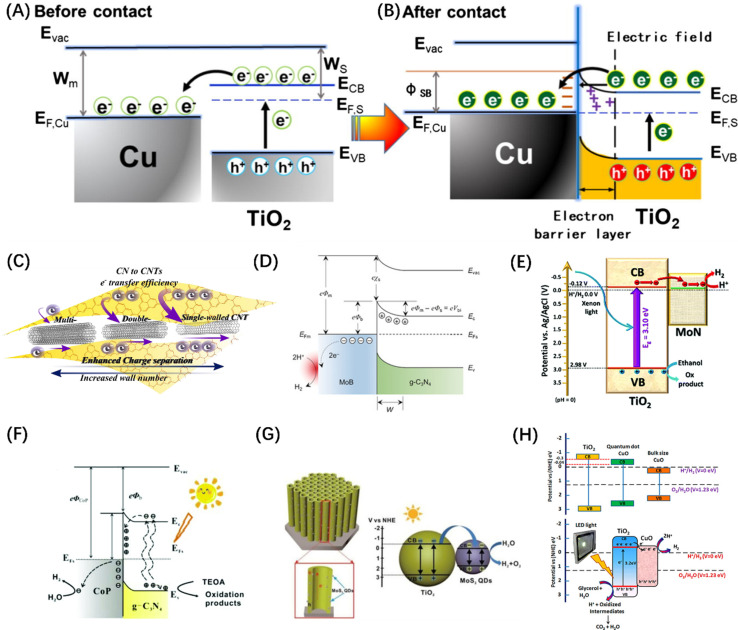
The change of energy band before (A) and after (B) contact between Cu and TiO_2_ and the migration path of photogenerated carriers. (A and B) Reproduced from ref. 105 with permission from Elsevier, copyright 2019. (C) Schematic diagram of photogenerated excitons to carbon nanotubes in g-C_3_N_4_. Reproduced from ref. 106 with permission from Elsevier, copyright 2018. (D) Schematic diagram of Schottky junction formed by MoB in contact with g-C_3_N_4_. Reproduced from ref. 107 with permission from John Wiley and Sons, copyright 2018. (E) The exciton dissociation and migration in the MoN/TiO_2_ system. Reproduced from ref. 108 with permission from Royal Society of Chemistry, copyright 2018. (F) Schematic diagram of the Schottky contact on CoP/g-C_3_N_4_. Reproduced from ref. 109 with permission from Royal Society of Chemistry, copyright 2018. (G) Energy band structure and charge transfer mechanism of MoS_2_@TiO_2_ heterostructure. Reproduced from ref. 110 with permission from John Wiley and Sons, copyright 2018. (H) Reaction mechanism diagram of photocatalytic water splitting over CuO–TiO_2_ nanocomposites. Reproduced from ref. 111 with permission from American Chemical Society, copyright 2018.

The most reported HER co-catalysts are metals and their alloys, especially coinage metals, such as Cu, Au, Ag, Pt, *etc.*^38^ The work functions of these metals are generally large, thus after the metals contact with a semiconductor, the e^−^ on the semiconductor CB spontaneously migrates to the noble metal until the Fermi levels of the two sides of the heterojunction are balanced. At the same time, the h^+^ remains in VB, resulting in the bending of the semiconductor's energy band and the formation of a space charge region, which is responsible for the formation of the Schottky barrier, as shown in Fig. 7(A) and (B).^105^ For such a configuration, the metal can function as an electron trap to separate carriers, thus contributing to the improved photocatalytic activity. On this basis, an alloy co-catalyst formed by assembling two metals with different Fermi levels has a greater charge separation efficiency than a single metal, resulting in a synergistic effect of the two. Metal alloys, such as AuPd,^112^ NiMo,^113^ and NiCo,^114^ have been reported as photocatalysts for the HER. Similarly, carbon-based materials, such as carbon quantum dots,^115^ carbon nanotubes (Fig. 7C),^106^ graphene,^116^ and other carbon-based materials are frequently used to modify semiconductor base materials to enhance the HER activity because of their good conductivity and electron storage capabilities. It is worth mentioning that metal borides and metal nitrides can also integrate Schottky junctions in contact with semiconductors due to their metal-like properties.^38^ For example, VB_2_,^117^ MoB (Fig. 7D),^107^ NbN,^118^ and MoN (Fig. 7E)^108^ have been demonstrated to promote the transfer of photogenerated electrons in CdS and increase the evolution of H_2_; meanwhile, some co-catalysts can also enhance the stability of CdS. In addition, transition metal phosphides, such as FeP,^119^ CoP (Fig. 7F),^109^ Ni_2_P,^120^ and Cu_3_P,^121^ have been developed for photocatalytic HER, because of the “ensemble effect”. The P atoms in these phosphides have modest bonding strength to hydrogen, which facilitates the formation of H_2_ molecules while reducing the H-poisoning effect.^122^ Transition metal sulphides have also been extensively exploited as co-catalysts for photocatalytic HER due to their admirable surface properties which are well suited to the surface binding of both water molecules and H protons and their effective activity in boosting photogenerated exciton separation.^38^ Quantum dot (QD) materials, such as MoS_2_ (Fig. 7G),^110^ Co_3_S_4_,^123^ NiS_2_,^124^ and CuInS_2_ (ref. 125) show higher photocatalytic HER activity, particularly when smaller QD are employed. Some transition metal oxides, after reducing their size, can also serve as HER sites. Taking the TiO_2_ host catalyst as an example, CuO (Fig. 7H),^111^ Cu_2_O,^126^ and CoO^127^ can all be used as co-catalysts to foster the spatial separation of photogenerated carriers. Similarly, transition metal hydroxides such as Ni(OH)_2_ (ref. 128) and Cu(OH)_2_ (ref. 129) can also form heterojunctions with semiconductors to suppress the self-coupling of photogenerated carriers.

### 3.3 Co-catalysts for water oxidation

**Fig. 8:**
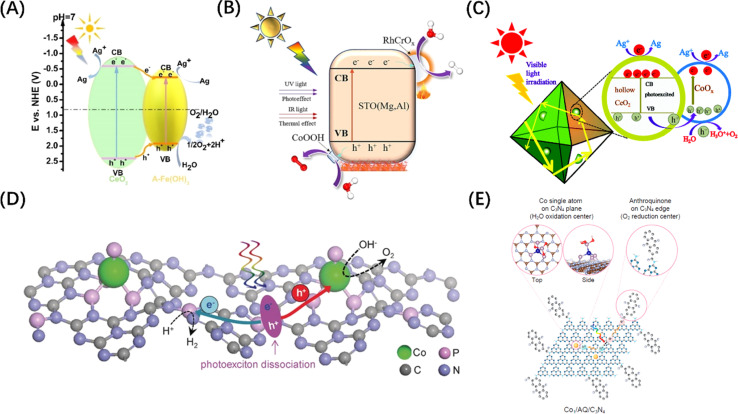
(A) Energy level comparison and carrier migration path diagram of CeO_2_/Fe(OH)_3_ heterostructure. Reproduced from ref. 131 with permission from John Wiley and Sons, copyright 2021. (B) Mechanism diagram of photothermal catalytic water splitting over SrTiO_3_ loaded with RhCrO_*x*_/CoOOH. Reproduced from ref. 132 with permission from Elsevier, copyright 2024. (C) Mechanism diagram of photocatalytic water splitting over CoO_*x*_-loaded CeO_2_ composite. Reproduced from ref. 133 with permission from Royal Society of Chemistry, copyright 2017. (D) Schematic diagram of POWS on Co–P-modified g-C_3_N_4_ photocatalyst. Reproduced from ref. 134 with permission from John Wiley and Sons, copyright 2017. (E) Schematic diagram of the redox sites on anthraquinone-loaded g-C_3_N_4_ embedded with Co single atoms. Reproduced from ref. 135 with permission from *PNAS*, copyright 2020.

Since the OER process consumes four h^+^ per two water molecules and is slower than the HER process, the function of the co-catalyst for H_2_O oxidation mainly involves two aspects: accelerating the transfer of h^+^ to the surface-active sites; and reducing the overpotential of the OER. Although metal co-catalysts can serve as e^−^ potential traps to promote the HER reactions and improve the OER activity to a certain extent, this indirect effect is limited by the lack of high-flux carrier catalytic sites. To address this problem, some hydrophilic hydroxides (Co(OH)_2_,^130^ Fe(OH)_3_ (Fig. 8A),^131^ Ni(OH)_2_,^136^*etc.*) or hydroxyl hydroxides (CoOOH (Fig. 8B)^132^ and FeOOH^137^) have been identified as ideal OER co-catalyst candidates. The VB potential of these co-catalysts is usually more negative than that of the host semiconductor. Therefore, h^+^ tends to migrate from the VB of the host semiconductor to the VB of the co-catalyst when the two materials are brought into contact, causing the energy band to bend to form a h^+^ barrier. There are also some transition metal oxides (CoO_*x*_ (Fig. 8C),^133^ MnO_*x*_,^138^ NiCo_2_O_4_,^139^*etc.*) used as HER co-catalysts that can also enhance OER if the VB is compatible with the host semiconductors. Of course, if the CBM of the co-catalyst is simultaneously more negative than that of the host semiconductor, the photogenerated carriers will be spatially separated to the greatest extent. It is worth mentioning that the physical size of co-catalyst is generally on the nanoscale, and the co-catalyst particles are highly dispersed over the bulk semiconductor, which ensures the formation of high-flux and high-density catalytic sites. In addition, some single-atom metals, such as Co (Fig. 8D and E)^134,135^ and Fe,^140^ can also enhance the OER activity. Different from the mechanism of metal enhancing the HER activity of host semiconductors, single-atom metals can form surface unsaturated coordination by combining with or replacing host atoms to form catalytic active sites.

## 4. Field promotion behind catalyst modification strategies

While co-catalyst modification can improve the surface reaction of a catalyst to a certain extent, the trace nature of the co-catalytic sites often results in limited promotion of the POWS process. Therefore, it is necessary to synchronize other intrinsic modification strategies, such as the manipulation of internal structure and energy fields to improve light absorption capabilities, enhancement of carrier dynamics, and promotion of charge output.^36^ In the sections that follow, the mechanisms of these energy field modifications are analysed from the perspective of improvements in these properties.

### 4.1 Light absorption enhancement

#### 4.1.1 Band engineering

The absorption of photons by a semiconductor is the first step in the POWS process and also serves as the energy source for subsequent carrier generation and reactions. Therefore, it is crucial to enhance the absorption of photons by semiconductors. To this end, there are generally two approaches, *i.e.*, expanding the spectral response range and upgrading the utilization of the entire light spectrum. According to the eqn (4), expanding the spectral response range can be realized by band modification for semiconductors to reduce the energy of the electron transition. Modification may include: metal doping; non-metal doping; and vacancy engineering.^141^

**Fig. 9:**
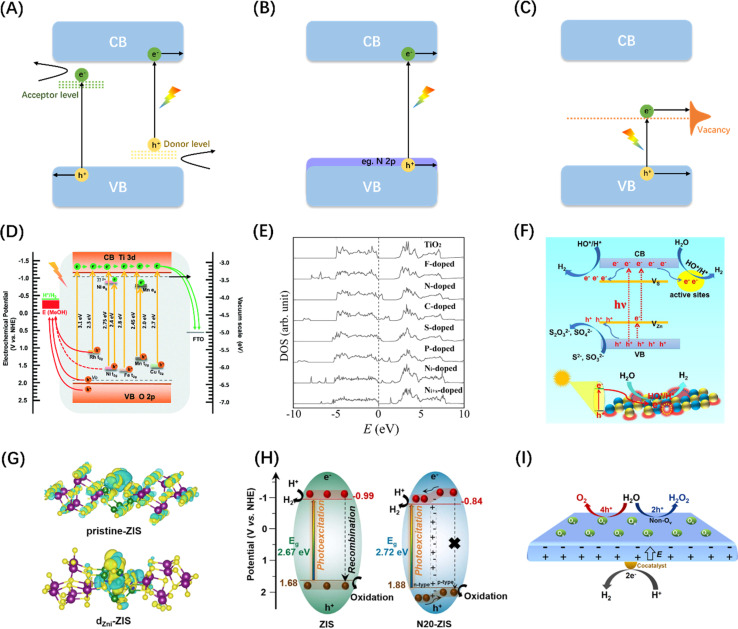
Schematic diagram of semiconductor energy band engineering: (A) metal doping; (B) non-metal element doping; (C) vacancy engineering. (D) Comparison of energy bands of SiTiO_3_ doped with different metal elements. Reproduced from ref. 143 with permission from Royal Society of Chemistry, copyright 2018. (E) Densities of states (DOSs) of the substitutional doping of C, N, F, P, or S for O in the anatase TiO_2_ crystal. Adapted from ref. 144 with permission from The American Association for the Advancement of Science, copyright 2001. (F) Diagram of photocatalytic water splitting and charge transfer mechanism in ZnS with abundant surface vacancy defects under sunlight irradiations. Reproduced from ref. 145 with permission from American Chemical Society, copyright 2021. (G) Differential charge density maps of the primordial-ZIS atomic layer and the d_Zni_-ZIS atomic layer. Reproduced from ref. 146 with permission from Elsevier, copyright 2022. (H) Schematic diagram of band structure and corresponding photoinduced activity of original ZIS and N-doped ZIS samples. Reproduced from ref. 147 with permission from Elsevier, copyright 2023. (I) Diagram of charge separation mechanism between different crystal faces of PbTiO_3_ caused by oxygen vacancy. Reproduced from ref. 148 with permission from American Chemical Society, copyright 2022.

Metal doping is a successful strategy for enhancing the spectral sensitivity of semiconductor photocatalysts possessing wide bandgap properties. The incorporation of metal cations possessing ionic radii comparable to those of the host ions may diminish the impetus for electronic transitions by either engaging with the VB or CB, or by creating energy levels within the band gap,^142^ which can change the physical properties of the intrinsic semiconductor so that it has p/n characteristics, as shown in the Fig. 9(A). Various metal ions, such as V^4+^, Cr^3+^, Mn^3+^, Fe^3+^, and Ni^2+^, were introduced into TiO_2_ to promote OER and/or HER reactions under visible light irradiations.^149^ A similar strategy of enhancing water splitting performance by metal doping was also demonstrated in a study of SrTiO_3_ material, as shown in Fig. 9(D).^143^ Combined with relevant computational studies, the optical response shift after doping is related to the ionic radius of the dopant. The change in the absorption edge typically rises as the cation radius decreases, while the energy states of impurities tend to shift towards lower energy levels as the atomic number of the dopant increases.^150^

Unlike metal doping that introduces interband energy states, doping by introducing a trace amount of non-metallic N, C, S, or halogen (F, Cl, Br) into the semiconductor structure generally reduces the band gap by increasing the VB edge (Fig. 9B).^151^ Since N_p_ orbitals are well suited to mix with O_2p_ and the ionic radii of the two elements are similar, N doping is most effective for oxide semiconductors (Fig. 9E).^144^ In addition, studies have found that introducing vacancies in semiconductors can also generate intermediate state energy levels, which can not only reduce the activation energy of photoelectrons but also serve as surface active sites to promote photocatalytic reactions (Fig. 9C and F).^145,152^ Although band engineering can reduce the activation energy of electronic transitions to a certain extent, it will also reduce the redox potentials of the photogenerated charges. It is therefore critical to weigh the impact of the doping level on both the light absorbing properties and the redox potentials of the photocatalyst.

In addition to the function of adjusting semiconductor energy bands, doping and vacancy engineering have been shown in recent years to contribute to carrier separation by introducing internal electric fields. The general mechanism involves the internal polarization field caused by the inhomogeneity of the material's bulk phase/surface band due to local/unidirectional doping or vacancy defects, which is considered to be compatible with band gap regulation and local carrier separation. For example, Sun *et al.* prepared ultra-thin ZnIn_2_S_4_ nanosheets doped with *in situ* gap zinc (d_Zni_-ZIS), proving that *in situ* gap Zn doping can not only induce electrostatic potential difference in the nanosheets to accelerate the photogenerated carrier separation efficiency (Fig. 9G), but also widen the layer spacing and produce short-range disordered structures.^146^ Chong *et al.* synthesized nitrogen-doped ZnIn_2_S_4_ with double p/n charge properties and found that the substitution of S by external N atoms with different electronegativity and valence electrons ultimately improved the charge transport rate and inhibited electron–hole pair recombination due to local p properties caused by N doping (Fig. 9H), resulting in favourable charge redistribution.^147^ Wan *et al.* examined the effect of surface oxygen vacancy on the POWS performance of single-domain PbTiO_3_, and showed that the internal electric field between the negatively polarized crystal plane (001) with rich oxygen vacancy and the positively polarized plane can effectively promote charge separation (Fig. 9I).^148^ More interestingly, He *et al.* achieved POWS by introducing sulphur vacancy defects in the CdS body phase, which results from the acceleration of carrier transport kinetics from the body phase to the surface redox site due to the spin polarization field induced by the single atom S vacancy in the opposite direction of the Coulomb field.^153^

#### 4.1.2 Morphology engineering

It is well known that when light strikes a “black” solid material, absorption and diffuse reflection occur. To improve the utilization of light, increasing the light-receiving area or reducing reflection has naturally become the direction for researchers to design the morphology of materials. The history of designing the morphology of semiconductor photocatalysts has developed from the beginning of nanoparticles to 1D nanorods,^154^ nanowires,^155^ nanofibers,^156^ and then to 2D nanosheets,^157^ and even the prevailing hollow microspheres,^158^ 3D hierarchical assemblies,^159^ and network skeleton structures.^160^ These various morphologies demonstrate the pursuit of high light utilization efficiency. In addition to increasing the specific surface area, the pores in the nanostructure can also cause light to be repeatedly reflected and scattered in the nanopores to increase the chance of contact between photons and the photocatalyst, thereby improving the light absorption performance.^161^ Fabricating such structures usually needs surfactants or templates in the particle growth phase, which will affect the catalytic activity. Also, the process of removing surface capping agents and templates, such as strong acid washing, high-temperature calcination, *etc.*, usually results in a certain degree of deformation to the original shape. Therefore, it is challenging to synthesize a material with both a well-defined and consistent structure and a high light harnessing capability.

**Fig. 10:**
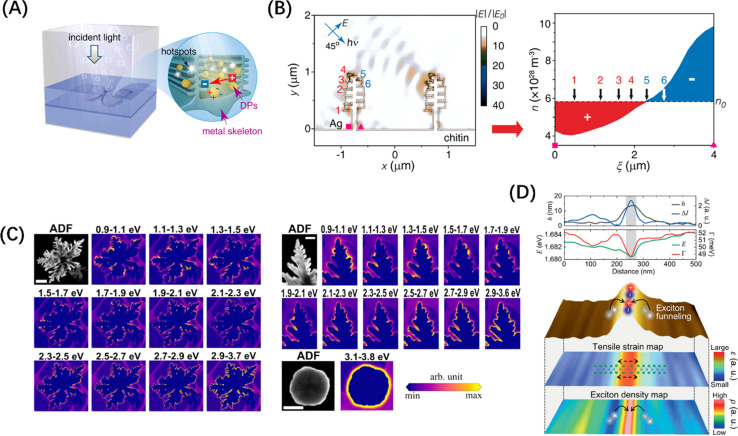
(A) Schematic diagram of photolysis of water by 3D Ag skeleton structure. (B) The simulated electrostatic force distribution in the 3D Ag skeleton and the electron density (*n*) distribution of the curved plate between the square and triangle symbols. (A and B) Reproduced from ref. 163 with permission from American Chemical Society, copyright 2016. (C) Annular dark field images of fractals (scale = 500 nm), branches (scale = 200 nm), and spherical particles (scale = 500 nm) with EELS intensity maps of their monomers. Reproduced from ref. 164 with permission from American Chemical Society, copyright 2021. (D) Line trace of WSe_2_ structure (top) and hyperspectral tip-enhanced photoluminescence spectrum and maps of expected tensile strain and exciton density near the fold (bottom). Reproduced from ref. 165 with permission from John Wiley and Sons, copyright 2021.

In addition to enhancing the frequency of interactions between incident photons and the material, a significant role of morphology regulation is to create centers for photogenerated electron concentration, which can disrupt the charge distribution among electron concentrations and distinct microregions, thus leading to the formation of a localized polarization field. Precious metal nanoparticles are a typical example, for them, the change in spectral response with geometry is only a symptom, and the change in the vibration behaviour of photogenerated hot electrons is the deep origin. For example, Ag nanotubes exhibit a wider light absorption range and a stronger local electric field than Ag nanospheres.^162^ Interestingly, Chen *et al.* achieved an electrical polarization of about 1.5 μm period through the asymmetric arrangement of plasma hot spots on the surface of the 3D Ag “nanotree” structure, and demonstrated by finite element simulation that the generated electric field can effectively separate the photogenerated hot electrons in the Ag “nanotree” skeleton (Fig. 10A and B), which is a strong proof of the microscopic long-range polarization field caused by topography control.^163^ Besides, Wang *et al.* designed and chemically synthesized dendritic Ag nanoparticles, and studied the spatial distribution of hot electrons at the single-particle level by electron energy loss spectroscopy (EELS), finding that the fractal with sharp tips and narrow gaps can support broadband resonances and many randomly distributed hot spots, and the enhanced field is mainly located around the individual protrusions of the “branch”, as shown in Fig. 10(C).^164^

This “tip effect” of field intensity distribution is also reflected in semiconductor materials. Koo *et al.* used hyperspectral adaptive tip enhanced PL spectroscopy to show the exciton distribution behaviour in different regions near naturally formed wrinkles in WSe_2_ monolayers and found that exciton funnel effect occurs at the apex of the fold, and the exciton density is the largest, which is a strong evidence for the formation of local polarization field (Fig. 10D).^165^ In the case of the specialized material C_3_N_4_, research has demonstrated that its light absorption capabilities can be enhanced through the distortion of the planar structure and symmetry of the carbon nitride layer.^166^ On this basis, Wang *et al.*^167^ transformed the geometric form of polyheptahedral imine (PHI) from hexagonal prisms to hexagonal nanosheets by improving the molten salt process, revealing the exact enhancement mechanism behind the performance improvement brought about by morphology modification, namely, the extension of carrier lifetime caused by the regulation of the field range between conjugated layers. Furthermore, Zou *et al.*^168^ successfully synthesized ultrathin twisted PHI nanoplates derived from PHI nanosheets. The optimized structural configuration significantly facilitated the n–π* electron transition, achieving an impressive apparent quantum efficiency of 17.3% for photocatalytic hydrogen production from water at a wavelength of 500 nm. These evidences underscore the importance of the deep-level field regulation mechanism in the absorption enhancement attributed to morphological modifications.

### 4.2 Enhancement of carrier dynamics

#### 4.2.1 Interface electrostatic/polarized field

**Fig. 11:**
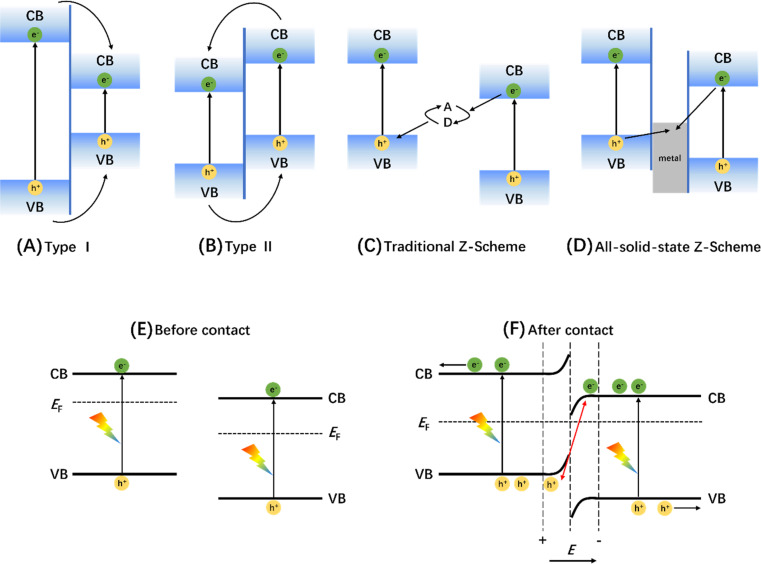
Charge-transfer route in (A) type I, (B) type II, (C) traditional Z-scheme, and (D) all-solid-state Z-scheme heterojunctions. Adapted from ref. 170 with permission from John Wiley and Sons, copyright 2017. Schematic diagram of energy band changes of semiconductors forming S-type heterojunctions before (E) and after (F) contact. Adapted from ref. 171 with permission from Elsevier, copyright 2020.

In a representative photocatalytic process, the photogenerated charge carriers migrate through the bulk to the surface where they interact with reactants. Aside from the impact of the degree of crystallization of the bulk catalyst, phonon scattering and bulk defects on carrier mobility and concentration, the recombination of charge carriers in the bulk caused by the electrostatic attraction between e^−^ and h^+^ is a common problem associated with single-material photocatalysts. The construction of heterojunctions can introduce local potentials at the photocatalyst interface that effectively stimulate the separation and improve the dynamics of photogenerated carriers. Currently, a variety of heterostructures have been developed, such as Type I, Type II, Z-scheme and S-scheme.^15,169^ The characteristic of Type I heterojunction is that e^−^ and h^+^ on the wide-gap semiconductor migrate to the CB and VB of the contacting narrow-gap semiconductor respectively. Although such migration process can assist charge separation, the redox potential of carriers is reduced, and the spatial concentration in the same semiconductor can easily lead to recombination (Fig. 11A). Compared with Type I, Type II heterojunctions have improved spatial distribution of photogenerated charges, but there is still a problem of redox energy loss (Fig. 11B). A variation of the Z-scheme heterojunction introduces a liquid phase redox mediator to consume the photogenerated charge carriers with a low redox capacity to retain the carriers on the high potential CB and VB (Fig. 11C). However, this kind of liquid-phase indirect Z-scheme heterojunction is rarely adopted due to the limitation of liquid-phase reaction, the side reaction of medium potential redox with high potential photogenerated charge, the influence of solution pH, and colored medium ions on the reaction and other factors.^170^ Therefore, metal-bridged Z-scheme heterojunctions, also called all-solid-state Z-scheme heterojunctions, were developed for liquid-phase or gas-phase reactions (Fig. 11D). The solid-state mediator not only reduces the transmission distance of electrons but also retains carriers at high potentials. However, creating a perfect semiconductor–metal–semiconductor bridge structure is difficult because point-to-point contact at the nano scale is difficult to achieve. Additionally, metals preloaded on the catalyst surface tend to behave as co-catalysts due to their small size and low content. After improvement, the Z-scheme heterojunction formed by direct contact between two semiconductors is also defined as the S-scheme heterojunction and has become a leading configuration in this type of photocatalyst. An S-scheme structure drives e^−^ and h^+^ at low potentials of the two semiconductors to self-annihilate through the built-in electric field at the contact interface, perfectly retaining the spatially separated photogenerated carriers at high potentials (Fig. 11E and F).^171^ As the most typical S-scheme heterojunction, WO_3_/g-C_3_N_4_ has been widely studied and applied to POWS since it was first reported.^172^ In addition, other systems, such as TiO_2_/MoO_3_,^173^ GaTe/PtS_2_,^174^ and PtS_2_/BN,^175^ have also been developed as catalysts with enhanced POWS activity. Some other heterojunction catalysts used for POWS in recent years are shown in Table 1.

**Table 1:** Typical heterojunctions for POWS

Material	Type	Co-catalyst	Reactant	Light source	Gas evolution rate (mol h^−1^ g^−1^)		Efficiency	Ref.
					H_2_	O_2_		
PCOS/Ni_2_P PCOS: polymeric carbon–oxygen semiconductor	I	NiS	Water containing MnO_2_	300 W Xe lamp *λ* > 420 nm	150.7	70.2	AQE = 70% at 420 nm, STH = 0.91%	176
P25/g-C_3_N_4_	II	Pt	Water	300 W Xe lamp	374.2	166	AQE = 0.71% at 400 nm	177
CdS/Ti^3+^–SrTiO_3_	II	MnO_*x*_	Water	300 W Xe lamp	176.07	86.03	AQE = 1.21% at 380 nm	138
Co_3_(PO_4_)_2_/g-C_3_N_4_	II	None	Water	300 W Xe lamp *λ* > 400 nm	375.6	177.4	AQE = 1.32% at 420 nm	178
ZnO/ZnS	II	Co_3_O_4_	Water	300 W Xe lamp 780 nm > *λ* > 320 nm	3853	1927	AQE = 3.04% at 350 nm	179
PbTiO_3_/BiVO_4_	Traditional Z	Rh/CrO_*x*_ Au/CoO_*x*_	Water containing Fe^2+^/Fe^3+^	300 W Xe lamp *λ* > 420 nm	48.04	24.19	STH = 0.053%	180
BiVO_4_/ZrO_2_/TaON	Traditional Z	Rh/Cr	Water containing K_4_ [Fe(CN)_6_]	300 W Xe lamp *λ* ≥ 420 nm	∼160	∼80	AQE = 12.3% at 420 nm, STH = 0.6%	181
g-C_3_N_4_/ITO/Co–BiVO_4_	All solid Z	Pt	Water	300 W Xe lamp AM 1.5 G	95.41	40.23	STH = 0.028%	182
BiVO_4_/Au/CdS	All solid Z	None	Water	Xe lamp AM 1.5 G	281	138	STH = 0.054%	183
In_2_Se_3_/CdTe	S	Pt/CoO_*x*_	Water	300 W Xe lamp *λ* ≥ 300 nm	101.15	47.38	STH = 1.31%	184
ZnIn_2_S_4_/WO_3_	S	Pt/CoO_*x*_	Water	300 W Xe lamp AM 1.5 G	169.2	82.5	STH = 1.52%	185
TiO_2_/ZnIn_2_S_4_	S	None	Water	300 W Xe lamp	214.9	81.7	AQE = 11.6% at 420 nm	186
InVO_4_/ZnIn_2_S_4_	S	None	Water	300 W Xe lamp	153.3	76.9	AQE = 9.75% at 420 nm	187

**Fig. 12:**
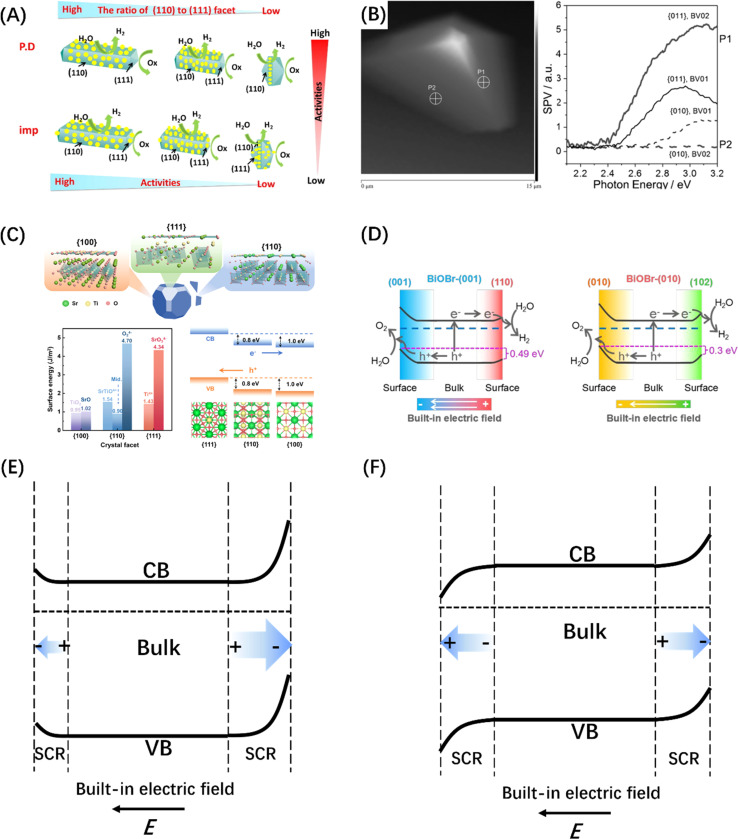
(A) Reduction and oxidation reactions take place on distinct crystal planes of TiO_2_, specifically on the (110) and (111) planes, respectively. Reproduced from ref. 190 with permission from Elsevier, copyright 2016. (B) The topological representation of a solitary BiVO_4_ crystal and spatially resolved surface photovoltage spectra obtained from various points. Reproduced from ref. 191 with permission from John Wiley and Sons, copyright 2015. (C) DFT studies of SrTiO_3_ crystallographic facets. Reproduced from ref. 192 with permission from American Chemical Society, copyright 2024. (D) Illustration depicting the process of charge separation induced by photogeneration in BiOBr-(001) and BiOBr-(010) surfaces, facilitated by the inherent electric field resulting from surface band bending. Reproduced from ref. 193 with permission from John Wiley and Sons, copyright 2020. Schematic diagram of the intensity anisotropy (E) and directional anisotropy (F) built-in electric fields on different crystal facets.

A built-in electric field can not only form at the interface by assembling two semiconductor photocatalysts, but also within a single semiconductor by engineering the crystal facet exposure of semiconductor crystal materials.^188,189^ The most typical example is that the (110) and (111) crystal facets of rutile TiO_2_ that have been identified as the sites of reduction and oxidation reactions, respectively (Fig. 12A).^190,194^ Subsequently, BiVO_4_ decahedral-shaped crystals have been favored by researchers due to its excellent photoanisotropy and suitable energy band structure; the e^−^ and h^+^ separation and accumulation over different crystal facets of a single BiVO_4_ crystal was also confirmed by Kelvin probe force microscopy (Fig. 12B).^191^ Besides, SrTiO_3_ crystals (Fig. 12C) with a narrower band gap and BiOX single crystals (Fig. 12D) that can control the surface atomic coordination of bismuth halide oxide by adjusting the configuration between the [Bi_2_O_2_] unit and the halide atoms have also been developed for crystal face control engineering and to introduce the built-in electric field.^192,193^ It was discovered that the directional separation of photo-induced carriers on different crystal facets is attributed to the difference in space charge regions caused by the different degrees of energy band bending at the surfaces of different crystal facets,^195^ manifesting in two aspects, *i.e.*, the difference in width and direction of the space charge region, corresponding to the difference in built-in electric field strength and direction respectively (Fig. 12E and F).

**Fig. 13:**
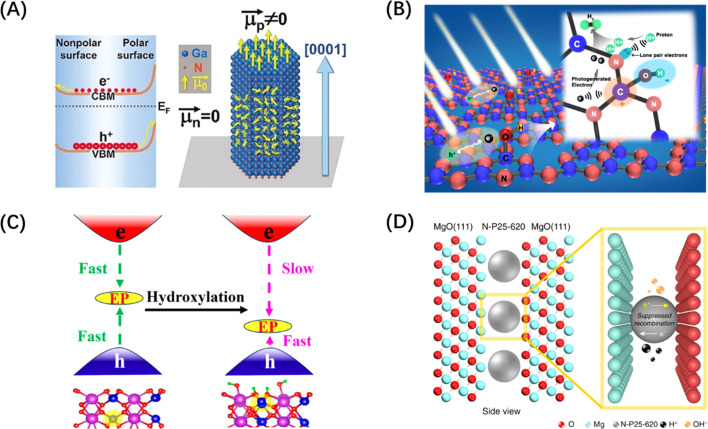
(A) Diagram of different band bending on polar and non-polar surfaces in a GaN crystal. Reproduced from ref. 198 with permission from John Wiley and Sons, copyright 2019. (B) Schematic diagram of charge separation of carbon nitride with surface grafted polymerized hydroxyl group. Reproduced from ref. 199 with permission from Elsevier, copyright 2018. (C) Schematic diagram of hydroxylation alleviating the harmful effects of electron polarons (EP) induced by oxygen vacancies in BiVO_4_. Reproduced from ref. 200 with permission from American Chemical Society, copyright 2023. (D) Schematic diagram of a polar MgO (111) nanocrystal with two electrical properties applying local electric field restriction to TiO_2_ nanoparticles. Reproduced from ref. 201 with permission from Springer Nature, copyright 2019.

Similarly, the polarization field based on the breakdown of the central symmetry of the crystalline material can also introduce an internal electric field. The direction of the polar surface means that each individual repeating unit grows in a direction perpendicular to the surface, causing the dipole moment of the surface to be non-zero, thus resulting in the generation of electrical and electrostatic power on the polar surface.^196^ Since the rigidity of some crystals prevents them from using the outer facet to introduce compensating charges to counteract the occurrence of polarity, the presence of macroscopic dipoles can leave considerable polarity and electrostatic force on the polar surface of such crystals.^197^ The underlying reason for the formation of this electrostatic force may be attributed to the difference in surface band bending caused by different surface polarities, which has been confirmed by the relevant studies on GaN single crystal arrays (Fig. 13A).^198^ In addition to the polarization field introduced by the selective growth of the polarized surface, surface polarization can also be induced by modifying polar groups, such as hydroxyl groups, on the catalyst surface. For example, the formation of H–O–C=N after the hydroxylation of g-C_3_N_4_ surface can foster electron movement to the surface –OH and accelerate the space charge separation, as shown in Fig. 13B.^199^ The hydroxylation of the surface of BiVO_4_ (010) can greatly reduce the negative effect of the electron polaron induced by oxygen vacancy and promote the splitting of water (Fig. 13C).^200^ It is worth mentioning that ferroelectric materials, mainly PbTiO_3_,^202^ have an internal electric field induced by spontaneous polarization because of their spontaneous dipole moment, which has also been proven to contribute to the self-separation of photogenerated carriers. Different from the spontaneous polarization of the above materials, confining the catalyst between two polarized crystal facets can also generate a polarization field and achieve the separation effect of photogenerated carriers. Such typical polarized crystal facets include MgO (111), NiO (111), and ZnO (0001).^203^ For example, the confinement of N-doped TiO_2_ nanoparticles between the positive and negative terminal surfaces of MgO (111) crystal faces can significantly improve the photogenerated carriers' lifetime (Fig. 13D).^201^

#### 4.2.2 Internal thermal gradients

**Fig. 14:**
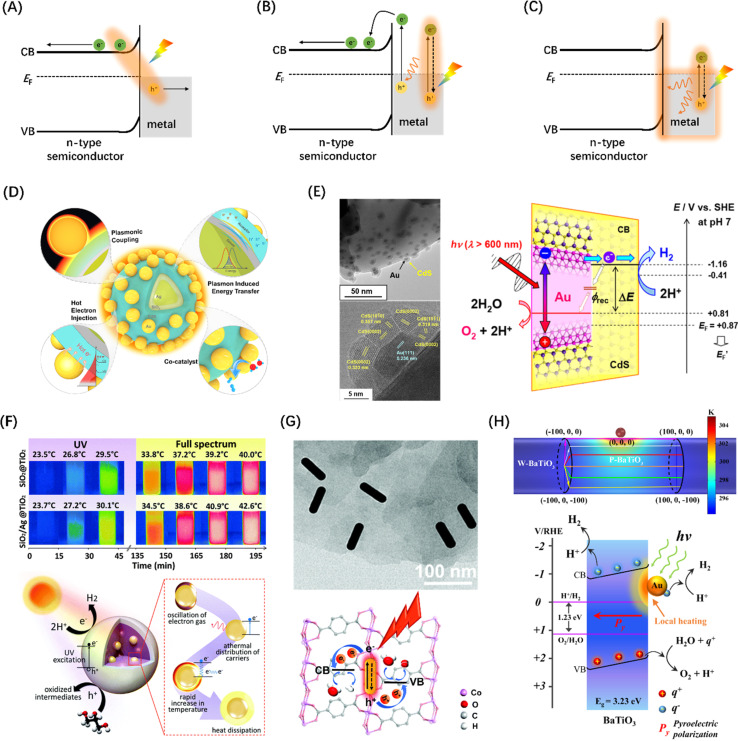
Metal-to-semiconductor charge–separation pathways: (A) plasma-induced direct charge transition at metal–semiconductor interface, (B) the light-excited plasma in the metal decays into hot electron–hole pairs by Landau damping, and (C) thermal effects of plasma decay through ohmic damping. (D) The role of plasma metals in different parts of core-shell-satellite photocatalysts in catalytic reactions. Reproduced from ref. 206 with permission from American Chemical Society, copyright 2021. (E) TEM and HR-TEM images of Au@CdS/ZnO composite structures and LSPR-induced hot electron transfer mechanism. Reproduced from ref. 207 with permission from American Chemical Society, copyright 2018. (F) Infrared images of containers dispersed with SiO_2_@TiO_2_ and SiO_2_/Ag@TiO_2_ nanocomposites taken under different light sources over time and the corresponding photothermal catalytic mechanism diagram. Reproduced from ref. 208 with permission from Royal Society of Chemistry, copyright 2016. (G) TEM characterization of AuNR/Co-MOFs composites and schematic illustration of HER and OER promotion mechanisms at AuNR/Co-MOFs composites upon light irradiations. Reproduced from ref. 209 with permission from Royal Society of Chemistry, copyright 2020. (H) Temperature distribution of a structural model of Au NP anchored to a BaTiO_3_ cylinder and schematic diagram of water splitting driven by local heating of a surface plasma. Reproduced from ref. 210 with permission from Springer Nature, copyright 2022.

The electron cloud in the metal particles tends to be shifted by the action of the photon wave when the size of the metal nanoparticles is coupled with the wavelength of the electric field of the incident light wave, thus leading to the formation of a pair of dipoles in a range of light wavelengths, which is “visually” viewed as plasma. This resonance effect of the electron cloud and the incident photon is called the surface plasmon resonance (SPR) effect.^204^ The photogenerated e^−^/h^+^ pairs caused by the SPR effect in metals will decay through radiative recombination and non-radiative recombination, where the former generates new photons, and the later mainly forms hot electrons.^205^ When there is a strong interdomain coupling between the metal and the semiconductor, the energy of the hot electron is high enough to overcome the Schottky barrier formed by the contact between the metal nanoparticle and the semiconductor, producing an electron on the semiconductor VB and leaving a hole in the metal (Fig. 14A). In addition, hot electrons can also stimulate the transition of electrons adjacent to CBM in the metal to the semiconductor by Landau damping (Fig. 14B),^211^ which is called Auger recombination. Otherwise, Shockley–Read–Hall recombination occurs, in which hot electrons transfer energy to the phonons of the adjacent semiconductor through ohmic damping and form an energy distribution within a certain range (Fig. 14C).^212^ Disturbance between phonons causes the crystal lattice to vibrate, leading to an increase in the local temperature of the semiconductor catalyst, which is beneficial to photocatalytic reactions. Extensive studies have proven that photo-thermal synergistic catalysis can significantly reduce the thermodynamic barrier of photocatalytic water splitting reactions, and the increase in temperature will accelerate the migration of photogenerated carriers.^213–215^ It should be noted that under widespread sunlight irradiations, metal nanoparticles play multiple roles in the photocatalytic water splitting reaction, depending on the constructed photocatalyst system and microstructure (Fig. 14D).^206^ For example, metal particles located inside the core–shell structure mainly contribute to the SPR photothermal effect, while those located on the surface are more likely to contribute to surface reactions.

A study proposed a half-cut Au@CdS core–shell structure, whose external quantum yield can reach 0.24% under 640 nm red light irradiations, originated from the injection of hot electrons generated by selective excitation in the Au core and their separation into the CdS conduction band, as shown in Fig. 14(E).^207^ Gao *et al.* designed a SiO_2_/Ag@TiO_2_ core–shell structure composite material as a solar thermal collector nanostructure, which has an efficient photothermal performance and can realize the synergistic reaction of seawater catalysis and desalination (Fig. 14F).^208^ Zhang *et al.* successfully achieved the simultaneous improvement of HER and OER by anchoring Au nanorods in Co-MOFs materials, and believed that the raising in activity was ascribed to the hot electron injection effect of Au nanorods into Co-MOFs (Fig. 14G).^209^ Creatively, You *et al.* introduced a local plasma heat source of Au nanoparticles into the thermocatalytic material BaTiO_3_, realizing that hot electrons can effectively trigger significantly accelerated multiple thermocatalytic cycle reactions (Fig. 14H).^210^ Moreover, the SPR effect is closely related to the micromorphology of the metal. So far, various novel morphological metal nanoparticles, such as nanocubes,^216^ nanorods,^217^ and nanostars,^218^ have been developed and used in metal–semiconductor systems for SPR-enhanced photothermal catalytic reactions.

### 4.3 Charge output promotion

**Fig. 15:**
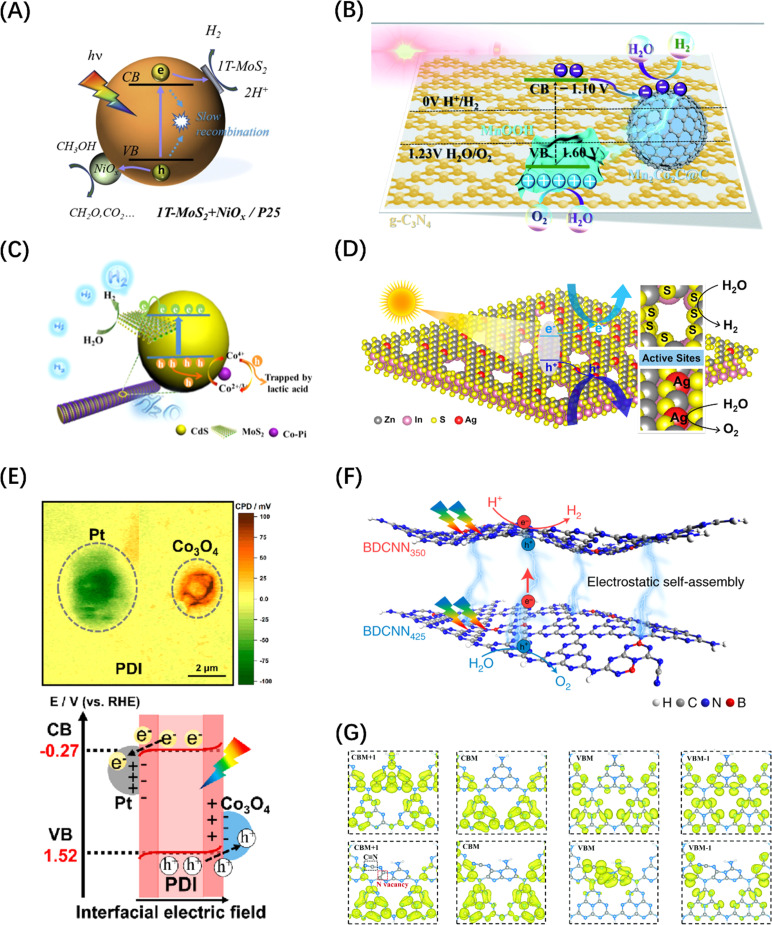
(A) Schematic diagram of carrier space separation in TiO_2_ enhanced by double co-catalyst MoS_2_ and NiO_*x*_. Reproduced from ref. 221 with permission from Elsevier, copyright 2020. (B) Schematic diagram of the mechanism of electron transfer and POWS in g-C_3_N_4_ promoted by double co-catalysts Mn_2_Co_2_C@C and MnOOH. Reproduced from ref. 223 with permission from Royal Society of Chemistry, copyright 2020. (C) HER photocatalytic mechanism diagram of MoS_2_ composites co-modified by CdS and Co-Pi. Reproduced from ref. 224 with permission from American Chemical Society, copyright 2019. (D) Schematic diagram of electrostatic field caused by the introduction of Ag doping and nanoporous defects on ZnIn_2_S_4_ monolayer. Reproduced from ref. 225 with permission from American Chemical Society, copyright 2021. (E) A mapping image of the contact potential difference between the double co-catalyst particles and the PDI film in the PDI/Co_3_O_4_/Pt heterostructure measured by Kelvin probe and a schematic diagram of the interface charge transfer path. Reproduced from ref. 226 with permission from American Chemical Society, copyright 2023. (F) Charge transfer diagram between g-C_3_N_4_ nanosheets with both B-doped and N-vacancy defects. Reproduced from ref. 227 with permission from Springer Nature, copyright 2021. (G) Calculated charge density distribution differences between CB and VB in PCN (top) and Nv–CN (bottom). Reproduced from ref. 228 with permission from Royal Society of Chemistry, copyright 2022.

Although the introduction of internal field can boost the dissociation and transport of photogenerated carriers within the catalyst, the photocatalytic reaction activity is largely affected by the surface reaction efficiency of the carriers. This surface reactivity is known to be mainly determined by the high-flux carrier accumulation sites and the catalytic reaction active sites, and when the two are spatially unified, the catalytic reaction efficiency will be doubled.^36,219^ Co-catalysts are usually modified on the catalyst surface to unify the two sites for POWS reactions. However, it is difficult for a catalyst to be modified with either a HER or OER co-catalysts to achieve overall water splitting, because the POWS process includes both reduction and oxidation processes, and which will affect each other if the high-flux sites and reactive sites are coupled, resulting in ineffective reactions. Therefore, the loading of dual co-catalysts is considered to be an effective modification scheme to achieve POWS.^220^ Many studies related to TiO_2_,^221^ C_3_N_4_,^222,223^ and CdS^224^ have confirmed that the dual co-catalyst scheme can not only accumulate two types of carriers in the corresponding reaction active sites but also separate the HER and OER reactions on the surface (Fig. 15A–C). Apart from the commonly used HER and OER co-catalysts, the introduction of vacancy defects and ion doping can also achieve the synergistic promotion of the two half reactions on the surface to achieve the effect of complete water splitting. For example, Pan *et al.* obtained a single-layer ZnIn_2_S_4_ nanosheet with double defects (Ag doping and nanopores) through cation exchange, which showed a stoichiometric H_2_ and O_2_ release in pure water under visible light irradiations (Fig. 15D).^225^

Briefly, the underlying logic of the dual promoter/defect modification to enhance the performance of POWS is still based on the enhancement mechanism of the built-in electrostatic field/polarization field, but there are different types of multi-directional energy field combinations in the same catalyst, such as heterojunction–heterojunction (electrostatic field-electrostatic field), heterojunction-defect/doping (electrostatic field-polarization field), or defect/doping-defect/doping (polarization field-polarization field), compared with a single heterostructure or doped/defect semiconductor. For example, Li *et al.* combined the dual co-catalysts Co_3_O_4_ and Pt with perylene diimide (PDI) polymer. The dual interfacial electric field constructed by the dual co-catalysts provided an anisotropic driving force for the photogenerated h^+^ and e^−^ in PDI, synergistically improving the spatial charge separation efficiency of water oxidation (Fig. 15E).^226^ Zhao *et al.* designed a Z-type system for photocatalytic water splitting based on boron-doped and nitrogen-defective carbon nitride 2D nanosheets, in which the doping of N and B not only promoted the carrier separation within the monolayer C_3_N_4_ plane, but also gave C_3_N_4_ different band structures, so that the interlayer electrostatic field was formed between the C_3_N_4_ nanosheets doped with the two different elements, as shown in Fig. 15(F).^227^ Zhang *et al.* introduced –C≡N groups and N vacancies into g-C_3_N_4_ (Nv–C N–CN) in sequence to cause the coexistence of double defects within the plane. Combined with the first-principle calculation results of the charge density distribution in the band structures of the original g-C_3_N_4_ and Nv–C N–CN, they proved that the charge density distribution corresponding to Nv–C N–CN is relatively separated when compared with the original g-C_3_N_4_, indicating that the modification of the double defect sites can lead to a more localized spatial distribution of the charge density (Fig. 15G), resulting in the improvement of the carrier separation ability.^228^

Nevertheless, it should be noted that the reactions occurring on the catalyst surface are very complex, specifically manifested as secondary reactions or cross-reactions of the products, which correspond to ORR reactions and reverse reactions in the POWS process.^49^ In order to avoid the occurrence of these two types of reactions, there are studies on extra modification of the catalyst surface, *i.e.*, surface coating engineering, finding that the photodeposited oxyhydroxide layer can act as a molecular sieve to selectively filter reactants and products.^229^ By utilizing the selectivity of the coating, the redox reaction on the photocatalyst surface can be appropriately controlled, leading to successful overall water splitting. However, the thickness and uniformity of the coating are difficult to control.

## 5. Macroscopic carriers and reaction kinetics

### 5.1 External energy field assistance

The geometric morphology control of photocatalysts, establishment of built-in fields, and surface modification all involve complex chemical synthesis processes, which limit the application of POWS technology. Especially in relation to the evaluation of POWS reaction efficiency, according to the solar hydrogen production (STH) efficiency standard:^230^5
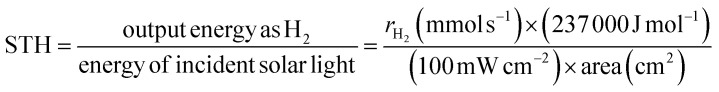
When the illumination area is fixed, STH is closely related to the apparent hydrogen evolution rate (*r*_H_2__).

Additionally, according to the POWS total conversion efficiency (*η*_total_) formula:^45^

6 *η*_total_ = *η*_absorption_ × *η*_separation_ × *η*_reaction_

The *η*_total_ is determined by three parts, namely, photon absorption efficiency (*η*_absorption_), carrier separation efficiency (*η*_separation_) and apparent reaction efficiency (*η*_reaction_). The first two can be adjusted by internal field modification, while the latter is mainly determined by the macroscopic reaction rate.

Utilizing external fields in photocatalytic reaction systems is a versatile and manageable approach to boost the apparent photocatalytic activity while keeping the properties of semiconductors unchanged. It has been reported that external field assistance, *e.g.*, external electric fields, thermal fields, and magnetic fields, can significantly improve the macroscopic reaction kinetics on photocatalysts, thereby improving the STH and *η*_reaction_.^231^

#### 5.1.1 Electric field

**Fig. 16:**
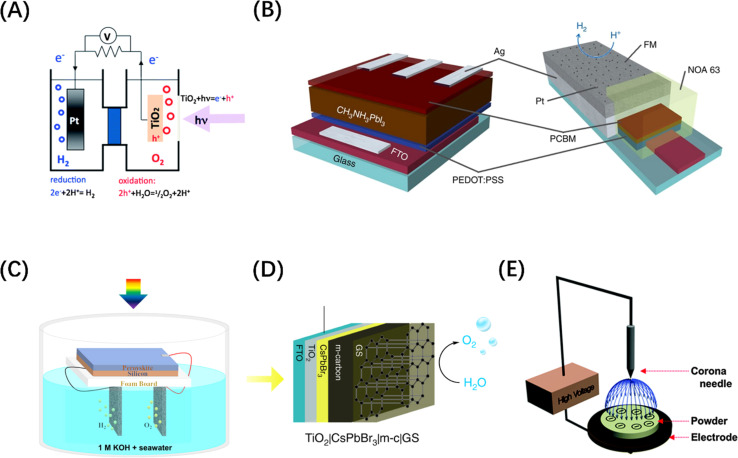
(A) Simple schematic of a photoelectrochemical cell. Reproduced from ref. 232 with permission from Royal Society of Chemistry, copyright 2014. (B) Diagram of a solar cell used as a photocathode for photocatalysis of HER. Reproduced from ref. 79 with permission from Springer Nature, copyright 2016. (C) Schematic diagram of the solar-driven seawater splitting device. Reproduced from ref. 233 with permission from American Chemical Society, copyright 2023. (D) Schematic of CsPbBr_3_-based multilayer photoanode for photoelectrochemical O_2_ evolution. Reproduced from ref. 234 with permission from Springer Nature, copyright 2019. (E) Illustration of the corona-poling system. Reproduced from ref. 235 with permission from Royal Society of Chemistry, copyright 2014.

The external electric field-assisted POWS process can be traced back to the origin of photocatalytic technology. The principle is to promote the separation of photogenerated carriers by applying an external bias voltage, thereby improving the solar energy conversion efficiency. Generally, the reaction loop of the liquid phase is similar to an electrolytic cell system, in which photogenerated carriers migrate to different electrodes under the force of external voltage, and then OER and HER reactions take place on the anode and cathode respectively, as shown in Fig. 16(A).^232^ Typically, high-performance photoelectrodes are fabricated using *in situ* growth techniques to establish intimate contact between the photocatalyst and the conductive substrate, a level of proximity that is challenging to attain with most particulate photocatalysts. Additionally, the efficacy of surface reactions within the photoelectrolyte is impeded by the extended migration distances and limited driving force of charge carriers. To overcome these challenges, both catalyst materials and photovoltaic systems have been developed.^236–238^ In terms of material selection, perovskite materials are favoured by researchers engaged in electrode-assisted POWS because of their excellent photoelectric response properties.^239^ At the same time, various optoelectronic reaction systems based on perovskite materials have been developed for POWS research, including external all-solid optoelectronic systems (Fig. 16B),^79^ seawater photoelectrolyte systems (Fig. 16C),^233^ multilayers (e^−^ transport layer/catalyst/h^+^ transport layer) photoelectrode systems (Fig. 16D),^234^*etc.* In addition, some ferroelectric materials exhibit spontaneous polarization and have bound charges on their surfaces, suggesting that the strong ferroelectric field formed can greatly promote the separation of body charges and surface charges in ferroelectric semiconductors under the driving of an external electric field.^240^ Interestingly, some studies have used corona technology to directly apply a strong electric field on ferroelectric powder samples to charge them to achieve a pre-polarization effect and achieve considerable improvement in the photocatalytic performances (Fig. 16E).^235^

#### 5.1.2 Thermal field

**Fig. 17:**
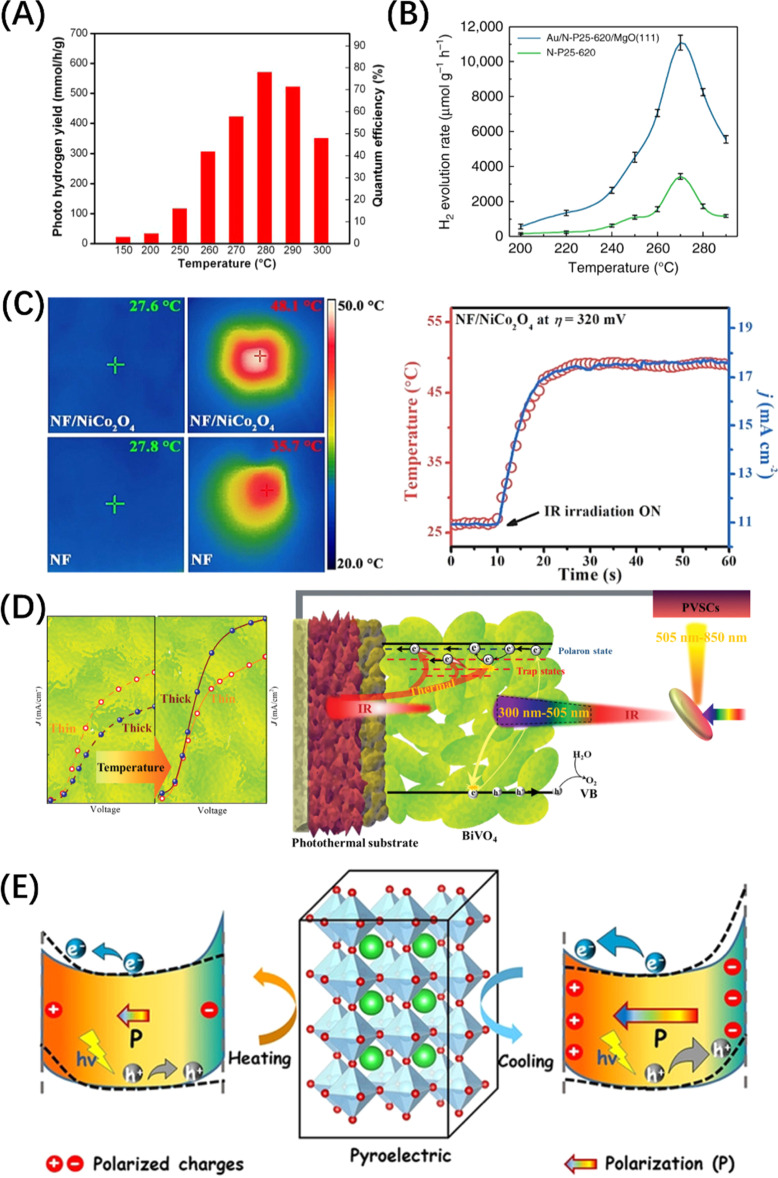
(A) Dependence of photocatalytic HER yield and apparent quantum efficiency on reaction temperature over Pt/black TiO_2_ catalyst. Reproduced from ref. 242 with permission from American Chemical Society, copyright 2015. (B) Temperature dependence of photocatalytic activity of N-doped P25 and Au/N-doped P25 confined by MgO (111). Reproduced from ref. 201 with permission from Springer Nature, copyright 2019. (C) Thermal images of NF/NiCo_2_O_4_ and NF in electrolyte solution under IR radiation and dark conditions and the dependence of temperature and current density *j* changes on IR radiation time. Reproduced from ref. 243 with permission from Elsevier, copyright 2023. (D) Schematic diagram of photocurrent difference of BiVO_4_ film with different thickness under light irradiations and schematic diagram of photothermally assisted water splitting. Reproduced from ref. 244 with permission from John Wiley and Sons, copyright 2022. (E) Schematic diagram of carrier separation behaviour and surface band bending differences in pyroelectrics debuted by the pyro-phototropic effect. Reproduced from ref. 231 with permission from John Wiley and Sons, copyright 2021.

The pyrolysis temperature of water is extremely high (over 1000 °C),^241^ making direct heating for water splitting impractical because of the high energy consumption. However, in a simple thermodynamic scheme, increasing the temperature is conducive to the forward progress of the endothermic reaction. Therefore, in theory, POWS can be promoted by heating. A study has induced Pt/TiO_2_ to photocatalytically split water in the presence of sacrificial agents by increasing the temperature (280 °C) and achieved an excellent quantum efficiency (Fig. 17A).^242^ Another study reported that N-doped TiO_2_ in the facet-limited domain of MgO (111) achieved excellent product release rates at 270 °C without sacrificing any reagents (Fig. 17B), and confirmed that the use of high temperatures can simultaneously increase the H^+^ and OH^−^ concentrations by increasing the hydro-ionization constant, suggesting a promising and effective method for promoting photocatalytic water splitting performance.^201^

Considering that infrared rays contribute nearly half of the solar spectrum, the required heat can be provided by infrared radiation, meaning that high-temperature-promoted photocatalysis can become a new scheme when the heat is provided by solar energy. A study of NiCo_2_O_4_ nanoneedles supported by nickel foam (NF) showed that the NF/NiCo_2_O_4_ class neural network structure acts as both an infrared absorbing antenna and an OER active anode and confirmed that the enhancement of OER activity is due to the local temperature increase under infrared radiations (Fig. 17C).^243^ Specifically, infrared radiation reduces the kinetic energy barrier of OER through the infrared thermal effect, thus promoting the OER dynamics. In addition to reducing the reaction thermal barrier, the thermal effect caused by infrared radiations can also stimulate the release of polarons in some interband defect levels and accelerate polaron energy level jumps (Fig. 17D).^244^ Besides, for a special kind of material, pyroelectric material, the introduction of temperature gradient can not only promote the surface reaction thermodynamically but also induce the generation of polarization field inside the material.^231^ The temperature-induced creation of positive and negative polarization charges at the extremities of the pyroelectric crystal can enhance charge separation efficiency and control photogenerated carrier transport at the interface, as shown in Fig. 17(E). The resulting thermoelectric potential can modify the surface charge energy, consequently initiating catalytic reactions through thermal catalysis and working in conjunction with the photocatalytic process to facilitate efficient photothermal catalytic reactions.

#### 5.1.3 Magnetic field

**Fig. 18:**
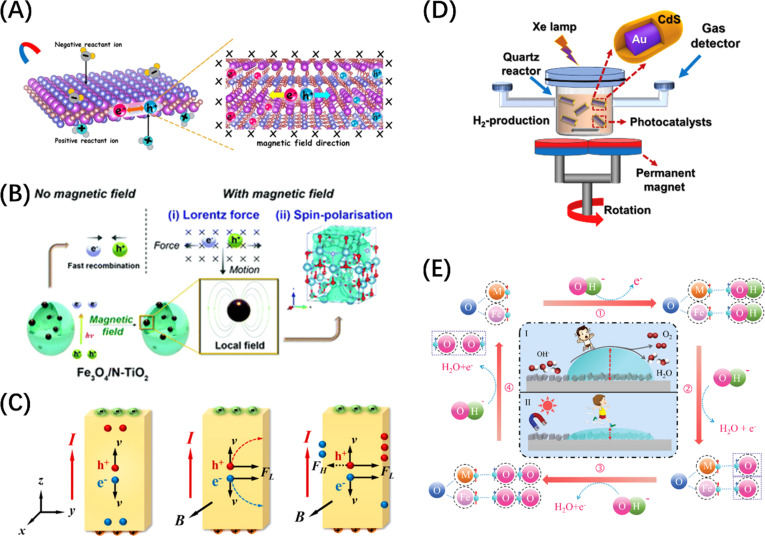
(A) Schematic diagram of macroscopic global charge separation assisted by external magnetic field. Reproduced from ref. 231 with permission from John Wiley and Sons, copyright 2021. (B) Schematic diagram of POWS performance of Au/Fe_3_O_4_/N–TiO_2_ composite material assisted by external magnetic field. Reproduced from ref. 246 with permission from Royal Society of Chemistry, copyright 2022. (C) Schematic diagram of the overall macroscopic charge separation caused by the change of the internal electric field in a polar photocatalyst under the action of the Lorentz force. Reproduced from ref. 23 with permission from American Chemical Society, copyright 2021. (D) Schematic diagram of an experimental setup showing the magnetic field-assisted photocatalytic water splitting system. Reproduced from ref. 247 with permission from Elsevier, copyright 2020. (E) Mechanism diagram of the combination of light and magnetic field to promote OER. Reproduced from ref. 248 with permission from John Wiley and Sons, copyright 2023.

Based on electron spin theory, electrons have two inherent properties: charge and spin, where spin includes two states: spin-up and spin-down. As per the Pauli exclusion principle, electron pairs occupying the same orbital exhibit opposing spins, resulting in counteracting magnetic fields and thus yielding a net spin current of zero. However, the introduction or extraction of a minor quantity of energy can induce a spin flip event, wherein the spin state of an electron is altered due to modifications in external magnetic fields or the absorption of light.^23^ This flipping of the electron spin state has been confirmed by many studies to have a positive impact on catalytic performance, which can be manifested in the enhanced interaction with reactant molecules, changes in conductivity and free energy, and the promotion of apparent kinetics.^245^ Moreover, the incorporation of a magnetic field can result in various changes, with one prominent effect being the utilization of the Lorentz force generated by the magnetic field as a propelling mechanism to enhance the segregation of photogenerated carriers and facilitate the attachment of ions onto the catalyst surface, as shown in Fig. 18(A). This, in turn, enhances the photocatalytic efficiency.^231^ An instance of this phenomenon is the utilization of an Au-supported Fe_3_O_4_/N–TiO_2_ superparamagnetic POWS catalyst, which has been observed to be enhanced by the local magnetic field effect. This catalyst is capable of generating a robust local magnetic flux through the application of a relatively weak external magnetic field measuring 180 mT. Consequently, it achieves a notable quantum efficiency of 88.7% at a wavelength of 437 nm under conditions of 270 °C, thanks to the combined effects of the Lorentz force and spin polarization, which serve to significantly prolong the exciton lifetime (Fig. 18B).^246^ In addition, for some polar catalysts, such as semiconductors with molecular dipoles, due to the existence of Hall effect in the electric field, static isoelectric focusing is easy to saturate both ends of the catalyst although it can play the effect of photo-generated carrier separation. As shown in Fig. 18(C), when the external magnetic field and the internal electric field of the material act on the *x* and *z* directions respectively, according to the left-hand rule, the Lorentz force on the carriers is in the same direction. This results in the same lateral movement of the hole and the electron, which can prevent the carriers from directly rushing to the centre of positive and negative charges, thus ensuring the continuous internal electric field effect.^23^ Of course, the performance of the catalyst in the magnetic field-assisted system is also closely related to the structure of the reactor. It has been reported that the efficiency of photocatalytic hydrogen production can be increased by about 110% by placing the core–shell nanostructures in ordinary photocatalytic reactors equipped with moving permanent magnets at the bottom (Fig. 18D).^247^

Although the field intensification technology has been developed to a great extent, the role of a single field is limited. It has been reported that the OER activity of Co_3_O_4_/CoFe_2_O_4_@NF composite photoelectrode materials with both optical and magnetic responsiveness can be improved by synergistically reducing the resistance and increasing the conductivity of the materials under the combined driving forces of optical and magnetic fields, as shown in Fig. 18(E).^248^ Besides, the electron polarization of the ferromagnetic catalyst under the magnetic field can reduce the potential barrier generated by parallel arranged paramagnetic oxygen and significantly improve the OER reaction kinetics. Therefore, it can be predicted that exploring the mechanism of photocatalysis driven by multi-energy field coupling will be the mainstream trend of future development, which can not only deepen the understanding of external field participation in photocatalytic reactions, but also make up for the possible limitation of a single external field.

### 5.2 Reaction type design and kinetics for scaling

In addition to modifying the properties of photocatalysts and using an auxiliary external field, it is also necessary to design and innovate the whole photocatalytic reaction to make the technology suitable for practical application. This is of great significance in improving the reaction efficiency and large-scale production research. The type of semiconductor POWS reaction has been developed from the initial electrode reaction to a variety of characteristic and practical systems, among which more common reactions are batch reaction, flow reaction, and gas–solid reaction.

#### 5.2.1 Batch reaction

**Fig. 19:**
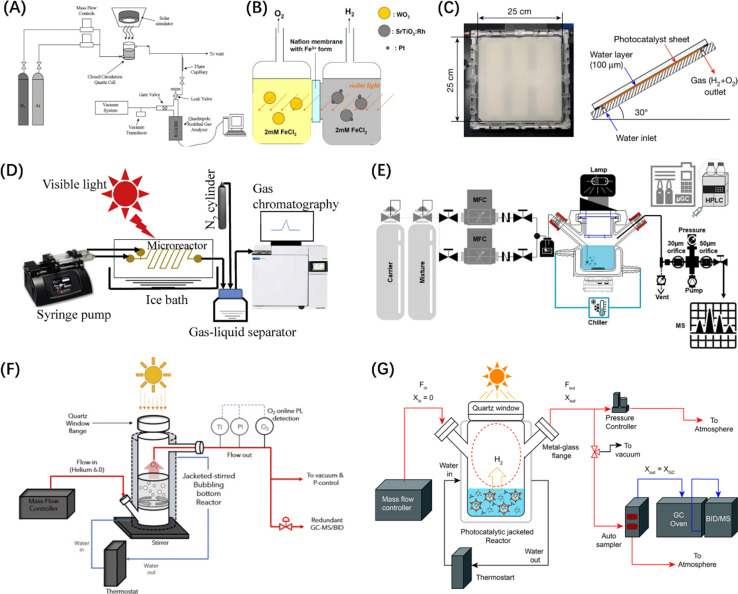
(A) Scheme of a batch water splitting reaction setup. Reproduced from ref. 250 with permission from John Wiley and Sons, copyright 2010. (B) Schematic diagram of a twin-reactor system. Reproduced from ref. 251 with permission from Elsevier, copyright 2010. (C) Photo image of the panel reactor unit and structural diagram of the panel reactor unit seen from the side. Reproduced from ref. 252 with permission from Springer Nature, copyright 2021. (D) Schematic diagram of an optofluidic microreactor for the photocatalytic water splitting. Reproduced from ref. 253 with permission from Elsevier, copyright 2022. (E) Diagram of a multimodal photocatalytic flow system. Reproduced from ref. 254 with permission from AIP Publishing, copyright 2023. (F) Schematic diagram of the evaluation system used to directly quantify the photocatalytic OER rate online. Reproduced from ref. 255 with permission from Elsevier, copyright 2020. (G) Illustration depicting the configuration of a continuous-flow photocatalytic reactor system. Reproduced from ref. 256 with permission from American Chemical Society, copyright 2019.

Batch reaction usually occurs within a solid–liquid reaction system. In a standard procedure, the reaction slurry suspension is contained within a reaction vessel constructed from materials such as stainless steel, Pyrex, or quartz. Throughout the reaction process, the slurry is thoroughly mixed by a magnetic stirrer to prevent catalyst particle deposition and agglomeration.^249^ The tank is usually equipped with a temperature control system so that the reaction process can be maintained at a certain temperature. Artificial light irradiates on the suspension through a quartz window on the reactor which is generally connected to carrier gas units, vacuum system, and gas collection/analysis instruments (Fig. 19A).^250^ To maximize the efficiency of batch reactions, it is particularly important to rationalize reactor design, which is related to photon utilization, the contact between catalysts and reactants, and mass transfer efficiency. Since the batch reaction was developed, various reactors emerged and are still being optimized and developed today. From the single reactor to the double reactor to hinder the complex reverse reaction of hydrogen and oxygen (Fig. 19B),^251^ from the initial cylindrical reaction tank to the panel reactor to expand the light area (Fig. 19C),^252^ from the general fluid reaction to the microfluidics to improve the energy density (Fig. 19D).^253^ These innovative improvements have contributed to the improvement of the overall photocatalytic efficiency. Although batch reaction systems have the advantages of low price, wide availability, and easy operation, they still have many disadvantages. The radiation field is inherently non-uniform, and there is no mechanical method to successfully mix photons that propagate at the speed of light, resulting in uneven distribution and attenuation of light in batch reaction systems, which leads to the reduced reaction efficiency.^257^ It is also difficult to control the atmosphere and temperature in batch systems due to mass transfer limitations. In addition, scaling up batch reactions will prolong the reaction time, which will hinder the rapid screening of target reactions.

#### 5.2.2 Flow type reaction

Flow reactions are similar to batch reactions, but the main difference is that batch reactors allow sufficient time for the reactants to react before achieving a cumulative yield, whereas in flow systems, the product is constantly removed from the reactor and tested online.^254^ Batch reactors are typically operated at lower pressures (below atmospheric pressure) and require the use of gas chromatography equipped with a thermal conductivity detector, resulting in a longer reaction time. The longer reaction time provides the possibility for the occurrence of reverse reactions or side reactions, which are extremely unfavourable to the photocatalytic efficiency. In addition, since the flow reaction system requires a certain pressure to ensure the continuous flow of gas throughout the reaction system, the reactor in the system is designed to have higher air tightness requirements. A multi-mode and multi-function flow reactor for characterizing powders and immobilized photocatalysts has been designed to quantify transient gas phase reaction products by online real-time gas analyser mass spectrometry (RTGA-MS), as shown in Fig. 19(E).^254^ The RTGA-MS gas detection sensitivity of this photocatalytic system spans three orders of magnitude for the most challenging gas H_2_, specifically, tens of parts per million can be detected under atmospheric conditions. The efficiency of the flow system also depends on the reactor design. For example, a research was conducted to accurately measure OER rates on P25-@RuO_2_ using a continuous flow high-purity glass reactor, which has a minimum detectable activity of 0.02 μmol O_2_ per hour, making it suitable for precise quantification of OER rates (Fig. 19F).^255^ Non-invasive direct H_2_ productivity monitoring through a specially designed continuous flow system has also been investigated, providing greater quantitative accuracy as compared to existing batch measurement methods (Fig. 19G).^256^

#### 5.2.3 Gas–solid phase reaction

**Fig. 20:**
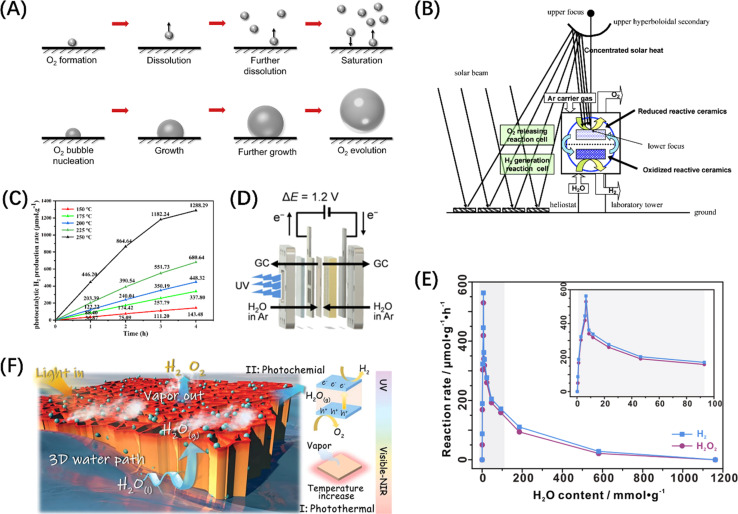
(A) Oxygen evolution diagram in POWS. Reproduced from ref. 49 with permission from Elsevier, copyright 2022. (B) A conceptual outline of a rotary solar reactor. Reproduced from ref. 258 with permission from American Chemical Society, copyright 2007. (C) Photocatalytic H_2_ production rate at different reaction temperatures over ZnTi. Reproduced from ref. 259 with permission from Royal Society of Chemistry, copyright 2022. (D) Schematic diagram of a gas–solid phase POWS battery. Reproduced from ref. 260 with permission from John Wiley and Sons, copyright 2018. (E) The progression of the observable photocatalytic efficacy in the complete splitting of pure water into H_2_ and H_2_O_2_ in relation to the water concentration on Pt/TiO_2_. Reproduced from ref. 261 with permission from Elsevier, copyright 2020. (F) Schematic diagram of photothermal catalytic water vapor splitting on TiN silicon wool loaded with K–SrTiO_3_. Reproduced from ref. 262 with permission from John Wiley and Sons, copyright 2023.

As the most direct reaction type, *i.e.*, solid–liquid reaction, it is still unable to break through a certain upper limit of efficiency, despite of the continuous improvement, including the expansion of new catalysts, the innovation of reactors, and the intervention of various auxiliary means. Moreover, due to the limitation of the liquid reaction temperature (usually less than 100 °C), it is difficult to make a breakthrough in the thermodynamic level of the reaction. At the same time, photon energy is wasted, especially the infrared radiation, which accounts for above 50% of solar energy. In addition, the formation of photocatalytic bubbles (H_2_ and O_2_) at the solid–liquid interface will affect the absorption capacity of the catalyst.^49^ Taking O_2_ as an example, due to the strong solubility of O_2_ in water, the initially formed O_2_ tends to be preferentially dissolved in the reaction solution. When saturated, free oxygen is released from the catalyst surface, as shown in Fig. 20(A). However, free oxygen has a high surface energy and tends to aggregate and be adsorbed on the catalyst surface. The light scattering and reflection on the bubble will reduce the intensity of light radiation and inhibit the exposure of the active site on the catalyst surface. In response to the above problems, researchers developed gas–solid POWS reaction types. It is reported that CeO_2_ and Ni, Mn-ferrite reaction ceramics can achieve a two-step water splitting process in a rotary solar reactor (Fig. 20B).^258^ Although many photothermal studies have confirmed that the efficiency of gaseous water splitting reaction at high temperatures is positively correlated with temperature (Fig. 20C),^259^ considering the energy consumption, some studies have also confirmed that relative humidity has a beneficial effect on water vapor splitting reaction in relatively mild conditions (Fig. 20D).^260,263^ For example, the surface catalytic activity of Pt/TiO_2_ to split water into H_2_ and H_2_O_2_ has a special functional relationship with the water load on the catalyst surface (Fig. 20E).^261^ Particularly, a study on coupling water evaporation and water splitting reactions at the water–air interface designed a TiN silicon wool material loaded with K–SrTiO_3_ (Fig. 20F), which can significantly reduce the reaction free energy of the catalyst by converting liquid water to water vapor, thereby increasing the transmittance of catalytic products.^262^ In general, the gas–solid reaction has the advantages of low free energy, high permeability of catalytic products, avoidance of liquid phase corrosion or dissolution, and easy recovery when compared with the solid–liquid reaction.

It is premature to assess the advantages and disadvantages of the three aforementioned reactor types solely from the standpoint of the production energy of the reactants, as not all catalysts are suitable for gas–solid reactions. Given the similarity in the energy field mechanisms across these reaction types, it is of practical importance to analyse the methods for enhancing efficiency from the perspective of the energy field. In macroscopic reactions, regardless of the type, the primary objective is to improve the light collection capability of the entire reaction system, which is fundamental to determining the efficiency of large-scale reaction systems.^4^ Research indicates that nearly half of the incident light is lost prior to being absorbed by the photocatalyst.^264^ Consequently, optimizing the parameters of the photon field—specifically, the intensity of light absorption and the distribution of photon flux—emerges as a critical factor in enhancing apparent reaction efficiency.

For typical liquid-phase intermittent reactions, which can generally be classified as suspension reactions, sunlight absorption is contingent upon the concentration of particles and the penetration depth of light.^4^ Therefore, identifying the optimal concentration of the reactive substance and increasing the light penetration depth are two significant strategies for improving macroscopic reaction efficiency. The former can be achieved through the parallel controlled experiments, while the latter can be realized through the optimization of reactor design. For instance, Goto *et al.* proposed a rectangular panel design for generating hydrogen and oxygen *via* a POWS reaction.^265^ This panel reactor features a water layer that is only 1 mm deep, facilitating the rapid release of product bubbles without the need for forced convection, while maximizing the inherent water splitting activity of the particle photocatalyst.

In contrast to batch reactions, the optimization of the photon field in liquid-phase flow reactions must also account for the effects of fluid dynamics.^266^ For example, Cao *et al.* introduced a gas disturbance reactor based on fluid dynamics principles. This photoreactor primarily operates under natural circulation, with high-pressure gas intermittently disturbing the deposited photocatalyst.^267^ By estimating parameters such as radiation distribution and critical flow rate, along with actual reactor commissioning, the necessity of gas perturbation was validated, resulting in optimal average hydrogen production rates of 2.9 L h^−1^ and 4.0 L h^−1^ during typical weeks in spring and summer, respectively. Furthermore, the nanofluid reactor presents a more compelling case. For instance, Zhang *et al.* demonstrated that the incorporation of BaTiO_3_ nanofluid into the POWS reaction significantly enhanced the hydrogen release rate, achieving a value of 270 mmol h^−1^ g^−1^.^268^ This improvement was attributed to the increased effective area for light irradiation and enhanced particle dispersion. It is evident that the gas–solid phase reaction system can mitigate the effects of suspended particle concentration and light penetration depth on the photon energy field to a certain extent; however, this does not necessarily imply the superiority, particularly in the context of the gas–solid POWS reaction.

The requirement for the temperature to exceed the boiling point of water imposes stringent demands on the temperature field distribution throughout the reaction. Concurrently, the optimization of the photon field must also be addressed, presenting significant challenges in reactor design. Due to the complexities associated with the temperature field control, current gas–solid reaction systems predominantly utilize fixed bed reactors, wherein the catalyst is immobilized within the reactor, such as thin-slit reactors, to maintain a uniform temperature distribution.^269^ In these systems, planar light is directed onto the catalyst surface *via* the optical fibre or waveguide technology.^270,271^ Consequently, it is essential to achieve effective coupling among the reactor, reaction medium, and photocatalyst particles across multiple physical fields to optimize light collection.

## 6. Conclusions, challenges and future perspectives

**Fig. 21:**
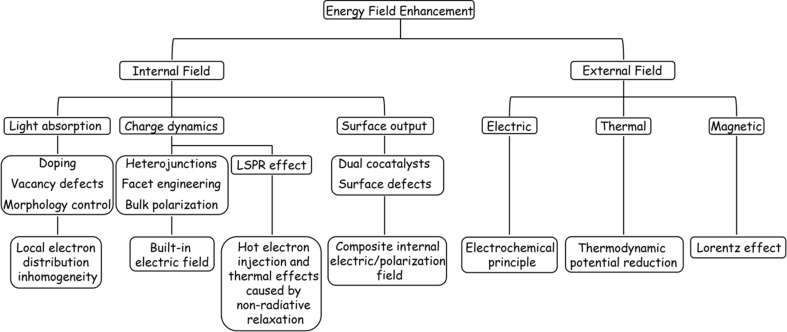
Energy field mechanisms behind the generalized POWS promotion strategies.

This paper reviews strategies to address the contemporary challenges in the POWS physicochemical process, focusing on microscopic and macroscopic modification methods for enhanced photogenerated charge carrier separation and promotion from the perspective of internal/external energy fields. Firstly, in view of the thermodynamic limitations of POWS, we examined the advantages and disadvantages of host semiconductor catalysts such as metal oxides, metal nitrides, metal sulphides, metal halide perovskites, carbon nitrides, and MOFs/COFs, as well as co-catalysts in POWS from the perspective of energy band modulation, combining the water splitting redox potential and the semiconductor band gap structure. Secondly, the mainstream semiconductor modification strategies contained in traditional modification concepts, for example, light absorption enhancement, carrier dynamics enhancement, and charge output promotion, including doping/vacancy engineering, heterojunction construction, crystal plane engineering, polarization engineering, hot electron decay and injection, and surface modification, are thoroughly analysed, and the underlying logic behind them, namely electrostatic field or polarization field, is thoroughly discussed. The role of an external field, such as external electric field, external thermal field, and external magnetic field, and different catalytic reaction systems, including batch reaction, flow reaction, and gas–solid phase reaction, are compared to discuss their improvement effects on macroscopic carriers and reaction kinetics. Finally, the strategies for enhancing the efficiency across various reaction types are critically reviewed from the viewpoint of the photon field. The field enhancement mechanisms corresponding to the modification strategies discussed in this paper are summarized in Fig. 21. Although there are numerous behavioural modifications of photogenerated excitons in POWS physical and chemical processes, there are still many challenges in this field at the overall level, such as the long-term time-consuming and uncertainty of experimental execution, the economic issues of catalytic materials, the instability and discontinuity of natural illumination, and the inefficiency of large-scale catalytic systems. Thus, based on the current efforts, we proposed the prospects for these four major challenges.

### 6.1 Utilization of machine learning *vs.* the long time and uncertainty of experimental execution

The development of POWS technology has been a topic of research for decades. Both the theoretical basis and practical application have been innovated, however the substantial improvement in efficiency only exists in the laboratory stage, and the economic benefits produced are still far from competing with traditional fossil energy technology. The economic and time costs associated with the development of high-performance materials and the interpretation of microscopic reaction mechanisms have greatly limited development through ‘trial and error’ based investigations. The integration of theoretical principles with practical experimentation is noteworthy for its ability to tackle the complexities associated with the advancement and enhancement of materials used in photocatalytic reactions. This approach serves as a foundational framework for devising innovative strategies to overcome technical obstacles. For example, DFT methods can been used to explore electronic problems, molecular dynamics simulations can describe interactions between large numbers of molecules and surfaces, Monte Carlo simulations can be used for even larger scale explorations, and continuum modelling methods are suitable for length scales close to experimental catalytic systems. Each approach makes a significant contribution to understanding the catalytic behaviour of materials. However, the actual POWS process is multi-length scale. Therefore, for the modelling of complex nanocatalysts, it is foreseeable that the future trend mainly involves combining methods, for instance, using machine learning, over different scales.

### 6.2 Development of new materials *vs.* economic issues of catalysts

In terms of material economic costs, there are also challenges. For example, precious metals and their oxides are considered to be the most active HER and OER catalysts, respectively, but their large-scale application is severely limited due to their scarcity and high cost. Although the material systems used for POWS have also been greatly expanded, including transition metal oxides, sulphides, nitride, perovskite materials, *etc.*, the development of cost-effective, earth-abundant, and high-performance catalysts remains a difficult task.

### 6.3 Introduction of energy storage systems *vs.* the instability and intermittency of natural sunlight

The intermittent nature of solar radiation is another objective challenge of POWS technology. Since solar radiation is only available during the day and sunshine also varies with cloud cover and seasons. As a result, the energy supply of a water-splitting reactor is subject to fluctuations of different periods and magnitudes. The resulting unstable operation results in low equipment utilization and reduced competitiveness with fossil fuels and nuclear energy. In order to achieve 24/7 continuous operation, current efforts are mainly focused on the efficient collection of full spectrum sunlight and the configuration of energy storage facilities combined with photothermal effects to maximize the use of photons to generate heat energy and extend the catalytic period after illumination.

### 6.4 Invention of new reactors *vs.* the inefficiency of large-scale catalytic systems

Promoting the large-scale use of POWS technology is the key to achieving its commercialization, thus it is necessary to develop safe and energy-efficient assembly reactors, which means developing simpler reactors made of lightweight and inexpensive materials but still ensuring safety and durability. At present, most of the research on large-scale POWS applications favours array panel reactors or composite parabolic condensers. To guarantee the consistent and dependable operation of extensive photothermal catalytic reactions, it is imperative to implement precise process control measures. This necessitates the integration of sophisticated monitoring and control systems that can effectively monitor crucial process variables and promptly modify operational settings as required.

## Data availability

No primary research results, software or code have been included and no new data were generated or analysed as part of this review.

## Author contributions

Wenhao Zhao: conceptualization, methodology, writing – original draft. Haijun Chen: resources, supervision. Jinqiang Zhang: methodology, writing – review & editing. Paul J. Low: supervision, writing – review & editing. Hongqi Sun: supervision, project administration, writing – review & editing.

## Conflicts of interest

There are no conflicts to declare.
